# Extraction of Curcuminoids and Carvacrol with Biobased Ionic Liquids—Evaluation of Anti-Cancer Properties of Curcuminoid Extracts

**DOI:** 10.3390/molecules30051180

**Published:** 2025-03-06

**Authors:** Chefikou Salami, Jean-Pierre Mbakidi, Sandra Audonnet, Sylvie Brassart-Pasco, Sandrine Bouquillon

**Affiliations:** 1 Institut de Chimie Moléculaire de Reims, UMR CNRS 7312, Université de Reims Champagne-Ardenne, Boîte n° 44, B.P. 1039, 51687 Reims, France; chefikou.salami@etudiant.univ-reims.fr (C.S.); jean-pierre.mbakidi@univ-reims.fr (J.-P.M.); 2 URCATech, URCACyt, Université de Reims Champagne-Ardenne, 51 Rue Cognacq Jay CS30018, 51095 Reims, France; sandra.audonnet@univ-reims.fr; 3 UMR CNRS/URCA 7369 (MEDyC), Université de Reims Champagne-Ardenne, 51 Rue Cognacq Jay CS30018, 51095 Reims, France; sylvie.brassart-pasco@univ-reims.fr

**Keywords:** ionic liquids, choline, curcuminoids, carvacrol, extraction, anti-cancer

## Abstract

Six biobased ionic liquids were prepared from saturated fatty acids (octanoic, decanoic and dodecanoic acids) and choline with yields up to 90% following procedures respecting green chemistry principles. These ionic liquids were fully characterized (NMR, IR, elemental analysis, viscosimetry and TGA) and used as extraction solvents for bioactive compounds (curcuminoids and carvacrol) using classical conditions, and the ionic liquids were able to be recovered after five runs without loss of activity. The ionic liquid containing a C12 carbon chain was the best extracting solvent, extracting 95% of the total curcuminoids contained in turmeric and 69% of the total carvacrol contained in oregano, which are higher yields compared to the extraction procedures described in the literature. As C12 ionic liquids were more cytotoxic than C8 ones, the biological activity of the curcuminoids extracted with C8 ionic liquids was evaluated on a MIAPaCa-2, a pancreatic adenocarcinoma cell line for which antitumor activity of curcuminoids had previously been reported. Compared to the cytotoxicity of the commercially available extract, the cytotoxic activity of the extracts was slightly weaker.

## 1. Introduction

Ionic liquids (ILs) consist entirely of ions (ammonium, phosphonium, imidazolium, pyridinium and sulfonium and mesylate, triflate, alcoholate or carboxylate (CH_3_CO_2_^−^, CF_3_CO_2_^−^ …) or inorganic ions (PF_6_^−^, BF_4_^−^, NO_3_^−^, Br^−^, Cl^−^, I^−^, …) [1,2]. They have interesting properties such as a melting point below 100 °C under 1 atm, low vapor pressure and physical and chemical stability [3]. Ionic liquids have a high dissolution and solvation capacity, which varies according to the nature of the cations and anions, making them excellent candidates as possible green substitutes for organic solvents in chemical reactions, extractions and biotransformations [4,5,6]. They are non-volatile, thermally stable and have a composition-dependent viscosity. Their density varies between 1.05 and 1.36 g/mL. The disadvantages of ionic liquids derived from petrosourced cations (imidazoliums, pyridiniums, etc.) are their high cost, poor biodegradability, biocompatibility and durability, hence the interest in synthesizing and using biosourced, biocompatible and biodegradable ionic liquids.

Ionic liquids (ILs) are of interest for a wide range of applications in chemistry, electrochemistry and biotechnology from a sustainable perspective [7]. Indeed, some of them could meet the needs of a more sustainable chemistry, which recommends the use of less polluting solvents and auxiliaries. In particular, ILs can be used in processes for treating biomass such as lignin [8] in the pharmaceutical and cosmetics industries [9], but also in the treatment of nuclear waste and the recovery of nuclear fuel in the electrochemical field [10]. Since some ILs can be immiscible with other solvents, they can form biphasic systems, and since organic species have a high solubility in these ILs, they are suitable as solvents for the extraction of most bioactive compounds from plants. For example, a hydrophobic IL based on butylmethylimidazolium and tetrafluoroborate (BMIM-BF_4_) forms a biphasic system in the presence of water for the extraction and separation of bioactive compounds such as phenolic compounds like piperine and tetrandine extracted from natural plants like *Lonicera japonica*, *Stephania tetrandra* and white pepper [11].

Choline-based ionic liquids (ChILs) are a class of ionic compounds that have gained significant attention in recent years due to their unique properties and potential applications in pharmaceutical formulations (green surfactants [12,13,14] biomass pretreatments (dissolution of lignin, cellulose and keratin [12,15,16,17] and catalysis (one pot three-component reaction, tricyanovinylation of indoles and Knoevenagel condensation for examples) [12,18,19,20]. Choline, a quaternary ammonium compound, is a naturally occurring substance found in many living organisms, where it plays a crucial role in various biological processes [21], being a source of the methyl groups needed to make the primary methyl donor S-adenosylmethionine, a part of the neurotransmitter acetylcholine, and a component of the major phospholipids in membranes. When combined with different anions, choline forms a variety of ChILs that exhibit excellent ionic conductivity, high thermal stability, and tunable physicochemical properties [22].

A few years ago, our group developed various ILs based on ammonium or phosphonium with anion from natural acids (L-lactic, L-tartaric, pyruvic, malic, malonic, succinic and osidic acids), but also from L-proline and its derivatives. These compounds showed in general a lower toxicity towards various organisms than usual chlorinated and commercial ILs [23,24,25]. Other ILs based on betaine [26] or choline [27,28,29] have also been prepared in recent years. In the case of choline derivatives, cholinium esters with saturated carbon chains have been prepared. The ecotoxicity of all these ILs was determined using a hemocyte-based bioassay [26] revealing the importance of the nature of both cations and anions in decreasing the ILs toxicity.

**Figure 1:**
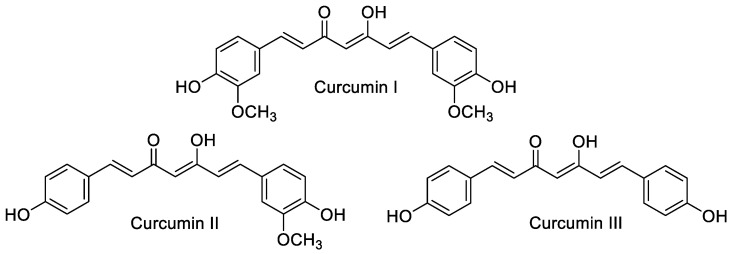
Curcumin structures.

Turmeric is widely cultivated in China, India and Southeast Asia. Turmeric powder has been employed in traditional Chinese medicine for more than 4000 years for the treatment of various ailments, including diabetic ulcers, coughs, liver disorders, gallbladder issues, sinusitis, rheumatoid arthritis, and loss of appetite [30]. According to Ayurvedic principles, turmeric boosts the general vigor of the body, enhances digestion, regulates menstrual cycles, dissolves gallstones, and relieves arthritis [31]. The therapeutical properties of turmeric are mainly attributed to curcumin (Curcumin I) due to its high concentration (75–80%) in turmeric extract in comparison to demethoxycurcumin (Curcumin II) (15–20%), bisdemethoxycurcumin (Curcumin III) (3–5%) [32] and cyclocurcumin (<1%) [33] (Figure 1). Bisdemethoxycurcumin possesses anti-oxidant, anti-cancer and anti-metastasis activities. Demethoxycurcumin has anti-inflammatory, anti-proliferative activities and is a candidate for the treatment of Alzheimer’s disease [34]. Cyclocurcumin was reported to prevent oxidative stress, rheumatoid arthritis and cardiovascular disorders [33]. Curcumin, which is the main coloring agent in the rhizomes of turmeric, has as anti-bacterial, anti-inflammatory, anti-proliferative, anti-metastatic, anti-angiogenic, anti-diabetic, hepato-protective, anti-atherosclerotic, anti-thrombotic, wound healing, anti-arthritic, neuroprotective, analgesic, immunomodulator and pulmonoprotective properties [35].

Curcuminoids also display anti-cancer properties, especially in pancreatic adenocarcinoma (PDAC) [36], which represents a significant public health concern, with both incidence and mortality rates steadily rising worldwide [37]. Curcuminoids have been categorized by the US FDA as “generally safe”, as they did not show significant side effects. Curcuminoids do not show any adverse effects up to a daily dose of 8 g to 12 g [38,39]. Despite many advantages, curcuminoid pharmacological efficacy is compromised in vivo due to poor aqueous solubility, poor gastrointestinal absorption, chemical instability, fast metabolism and quick systemic excretion that may result in poor systemic bioavailability [36]. In a Phase II clinical trial involving patients with advanced pancreatic cancer, an oral dose of 8 g curcuminoids per day resulted in plasma concentration of curcuminoids from 22 to 41 ng/mL [40]. Various nanotechnology-based delivery systems, such as nanotubes, nanofibers, micelles, liposomes, polymeric (e.g., PLGA [poly(lactic-co-glycolic acid)] and chitosan), protein (e.g., sunflower seed protein, bovine serum albumin), metal and solid-based lipid (e.g., long-PEGylated) nanoparticles have been found to increase curcuminoids bioavailability. Curcuminoids in combination with conventional chemotherapy drugs (doxorubicin, paclitaxel, 5-fluorouracil and cisplatin) have enhanced their therapeutic anti-cancer efficacy by targeting various molecules and regulating signaling pathways involved in cancer progression [41]. Moreover, curcuminoids were reported to overcome resistance and re-sensitize cancer to chemotherapeutic drugs in many studies [42].

Depending on its origin and the soil conditions in which it is grown, turmeric contains 2–9% curcuminoids, rising to around 20% depending on the season [43]. Curcuminoids are traditionally extracted from turmeric powder using polar organic solvents such as alcohols, acetone, ethyl acetate, etc. In 2010, Sogi et al. also studied the effect of four independent variables (temperature, particle size, mixing time and solvent (ethanol) to meal ratio on curcuminoid yield from turmeric (*Curcuma longa* L.)) [44]. Of all the solvents used, acetone gave the highest extraction yield (22.8%). The work of S.K. Bajpai et al. on the extraction of curcuminoids with 20 g of fine turmeric powder in 150 mL of acetone under moderate agitation for 72 h at 37 °C gave a yield of 14.95% by mass [45]. Just a little earlier, Pjo et al. studied the extraction of curcuminoids by using *s*CO_2_. They proved that curcuminoids were successfully extracted (31.07 mg from 1 g of curry powder over 2 h) with an ethanol modified *s*CO_2_ fluid (3.0 mL/min CO_2_ + 0.3 mL/min ethanol) at 60 °C with a pressure of 250 atm [46].

Finally, in 2018, Ranveer et al. showed that activation by ultrasound (25 W, 30 min) could increase the curcuminoid extraction yield by up to 6% [47]. Chen et al. used imidazolium and NTf_2_^−^ anion-based ILs as a curcuminoid extraction solvent through an ATPS-DLLME (aqueous two-phase extraction system—dispersive liquid-liquid microextraction) method; a 0.96% extraction yield with a purity of more than 51% with respect to the total dry mass of the product was obtained [48].

Thymol and carvacrol are among the compounds present in oregano (*Origanum vulgare*); this herbaceous perennial plant of the *Lamiaceae* family, an aromatic of the Mediterranean flora, is used in cooking and for medicinal purposes, as is its essential oil, which has anti-inflammatory properties. Its chemical composition can vary depending on the region; in Portuguese oregano, carvacrol, thymol, γ-terpinene and β-fenchylic alcohol are present as major compounds in large numbers [49].

Carvacrol extraction was carried out with 1 g of crushed oregano leaves in 100 mL EtOH (60%) at reflux 95 °C for 6 h with a yield of 0.75–7.5% carvacrol by mass for the species *Origanum savitum*. On the other hand, a yield of 2.75% was obtained by maceration for 12 h with the species *Origanum onites* [50]. Oliveira et al. have shown that oregano essential oil has a high carvacrol (38.6%) and thymol (18.5%) content, which varies according to species, growing location and season [51].

Carvacrol has been shown to have anti-proliferative activity on HepG2 hepatocellular carcinoma cell line with an IC50 = 0.40 mM [52]. Carvacrol also inhibits the growth of several bacterial strains, such as *Escherichia coli* and *Bacillus cereus* [53].

In the present study, we will prepare choline-based ionic ILs and perform the extraction of curcuminoids and carvacrol using those ILs. The extraction will be compared to previously described extraction results. Cytotoxicity of ILs will first be determined. Biological effects of extracted curcuminoids will then be explored.

## 2. Experimental Section

### 2.1. Chemicals Used

Choline (≥98%), levulinic acid (99%), lactic acid (85%), methanesulfonic acid (≥99%), octanoic acid (≥98%), dodecanoic acid (≥99%), sodium perchlorate (≥98%) and potassium lactate (60% in water) were purchased from Sigma–Aldrich (St. Louis, MO, USA). Choline was dried at 60 °C for 1 h prior to use. Other compounds were used as received. All aqueous solutions were prepared with distilled water.

### 2.2. Physicochemical Characterization

^1^H and ^13^C NMR spectra were recorded on an AC 500 Bruker for ^1^H and ^13^C spectra (500 MHz for ^1^H, 125 MHz for ^13^C, see Supplementary Materials). Chemical shifts (in ppm) for ^1^H and ^13^C NMR spectra were referenced to residual protic solvent peaks. IR spectra of liquid and solid compounds were recorded on a Bruker Alpha-T FTIR spectrometer at room temperature. Elemental analyses (C, H and N) were carried out on a Perkin–Elmer 2400 C, H, N, S element analyzer.

The direct infusion MS analysis was performed on a Waters Acquity UPLC system coupled with a Waters SYNAPT G2-Si High Resolution Mass Spectrometry equipped with electrospray ionization (ESI) source (Waters Corp., Manchester, UK). The mobile phase A consisted of water containing 0.1% formic acid, while the mobile phase B was acetonitrile. Mass detection was conducted in a negative ion mode, with the source temperature at 100 °C; capillary voltage and cone voltage were set at 2 KV (3 KV in positive mode) and 40 V. The desolvation gas was optimized to 650 L/h, the cone gas flow was 50 L/h and the scan range was from 50 to 1600 *m*/*z*. Mass was corrected during acquisition using external reference (Lock-Spray) consisting of a 1 ng/µL solution of leucine encephalin at a flow rate of 5 µL/min, in order to ensure the accuracy and reproducibility during the MS analysis. All data collected were acquired using MassLynx™ (V4.1) software in continuum mode.

Thermal decomposition of the studied ILs was conducted by heating samples of mass of several milligrams to 600 °C at a rate of 2 °C⋅min^−1^, using a NETZSCH TG 209 F1 Libra (Netzsch-Gerätebau GmbH Selb, Deutschland) recording microbalance in a stream of inert gas (Ar) at a flow rate of 50 mL·min^−1^. The sample was heated in an oven whose temperature was regulated by a programmable digital temperature controller. The carrier gas flow was controlled by electronic mass flowmeters. In addition to the TGA and DTG (first derivative of the TGA) curves, endothermic and exothermic effects were determined by means of the calculated DTA signal (c-DTA).

The viscosities measurements (in mPa·s) were performed using a Brookfield LV-DVII+ PRO viscometer with a CP51 cone spindle. The instrument was connected to a HUBER-ministat circulation-type thermo-regulated water bath, and measures between 298.15 and 353.15 K were realized. The repeatability of the viscometer was of 0.20% with an uncertainty in the viscosity measurements of 1.00% of the full-scale range, declared by the manufacturer.

To measure density at 298.15 K and constant atmospheric pressure, an Anton Paar oscillating tube densitometer DSA 5000 was used. The calibration of the instrument was performed using bi-distilled water and heptane. The standards and samples were degassed by sonication prior to measurements.

### 2.3. General Synthesis Process of Cholinium Ester-Based ILs Cho-C(8-12)-Lac and Cho-C(8-12)-Lev

The preparation of the choline based IL involved a three-step procedure (esterification followed by two anionic metathesis) with good yields (70–85%) according to procedures previously described [27,29] for compounds **4**, **5**, **7a**,**b** and **8a**,**b**.

The general procedure is outlined below [29]. The spectra of the compounds **4**, **5**, **7a**,**b** and **8a**,**b** are consistent with those described in the literature.

To obtain perchlorate intermediates Chol-Cn-ClO_4_, a suspension of dried choline (40 g, 286 mmol, 1.0 equiv.) in methane sulfonic acid (1.5 equiv.) reacted with an excess of hexanoic or decanoic acid (3 equiv.) at 100 °C under reduced pressure (50–100 mbar) to remove the water during 6 h. After addition of water (100–200 mL), the crude material was first washed with diethyl ether (5 × 100 mL) and then with dichloromethane (5 × 100 mL) to remove excess fatty carboxylic acid. Then, to eliminate excess unreacted choline, an excess of sodium perchlorate (3 equiv.), previously dissolved in a minimum of water (10–20 mL), was added to a solution of crude material in water. The mixture was stirred at room temperature for 24 h and the Chol-Cn-ClO4 was extracted in dichloromethane; after washing with water (5 × 100 mL) to remove excess sodium perchlorate and the free choline sometimes still present, the solvent was removed under reduced pressure, and the addition of diethyl ether precipitated the final compound. After filtration, the product was obtained as a white powder.

To obtain the lactate or the levulinate adduct, a slight excess of potassium lactate or levulinate (1.2 equiv.) in ethanol (20 mL) was added to a solution of Chol-Cn-ClO_4_ (1 equiv.) in ethanol (100 mL). The reaction mixture was stirred at room temperature for 24 h. After filtration of KClO_4_ and evaporation of the solvent under reduced pressure, the resulting IL was washed with diethyl ether and dried under vacuum for 3 days.

For the compound **7a** [29], the general procedure was used with 20 g of choline, 41.3 g of octanoic acid, 23.23 mL of methane sulfonic acid, 35.06 g of sodium perchlorate and 9.46 g of potassium lactate.


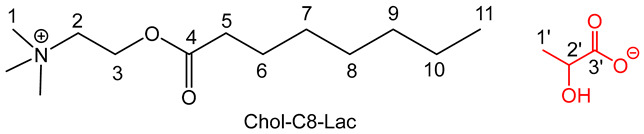

^1^H NMR δ ppm (500 MHz; DMSO *d*_6_): 0.86 (3 H, t, *J* = 7.5 Hz, H11), 1.07 (3 H, d, *J* = 7.5 Hz, H1′), 1.25 (8 H, H7,8,9,10), 1.54 (2 H, t, *J* = 7.5 Hz, H6), 2.34 (2 H, t, *J* = 7.5 Hz, H5), 3.16 (9 H, s, H1), 3.47 (1 H, q, *J* = 7.5 Hz, H2′), 3.71 (2 H, t, *J* = 7.5 Hz, H2), 4.45 (2 H, t, *J* = 7.5 Hz, H3) ppm. ^13^C NMR: δ ppm (62.5 MHz; DMSO *d*_6_): 14.1 (C11), 18.2 (C1′), 22.5, 24.5, 29.2, 29.3 (C 7,8,9,10), 31.6 (C6), 33.8 (C5), 53.2 (C1), 58.2 (C2), 64.1 (C3), 67.5 (C2′), 172.5 (C4), 177.8 (C3′) ppm. IR: ν (cm^−1^) 1116 (C-O-H Lactate), 1595 (C=O_Lactate_), 1737 (C=O_ester_), 2912 (N-CH), 2815 (CH_3_-N). Anal. calcd.: C_16_H_33_NO_5_: C 60.16; H 10.41; N 4.38%; found: C 59.64; H 9.98; N 4.32%.

For the compound **7b** [29], the general procedure was used with 20 g of choline, 41.3 g of octanoic acid, 23.23 mL of methane sulfonic acid, 35.06 g of sodium perchlorate and 12.2 g of potassium levulinate.


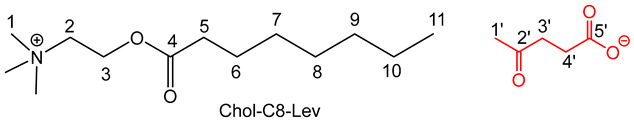

^1^H NMR: δ ppm (500 MHz, DMSO *d*_6_): 0.85 (3 H, t, *J* = 7.5 Hz, H11), 1.24 (8 H, m, H7,8,9,10), 1.53 (2 H, t, *J* = 7.5 Hz, H6), 2.06 (3 H, s, H1′), 2.21 (2 H, t, *J* = 7.5 Hz, H3′), 2.33 (2 H, t, *J* = 7.5 Hz, H3), 2.51 (2 H, t, *J* = 7.5 Hz, H4′), 3.16 (9 H, s, H1), 3.72 (2 H, t, *J* = 7.5 Hz, H2), 4.45 (2 H, t, *J* = 7.5 Hz, H3). ^13^C NMR δ (62.5 MHz, DMSO *d*_6_): 14.2 (C11), 22.4 (C1′), 24.2, 28.7, 29.2, 29.3, 29.3 (C 7,8,9,10), 32.4 (C6), 40.2 (C3′, C4′)), 53.1 (C1), 58.4 (C2), 63.1 (C3), 172.7 (C4), 176.6 (C5′), 208.9 (C2′) IR: ν (cm^−1^) 1580 (COO^−^), 1715 (C=O_ketone_), 1746 (C=O_ester_), 2902 (N-CH), 2820 (CH_3_-N). Anal. calcd.: C_18_H_35_NO_5_: C 62.58, H 10.21, N 4.05%; found: C 62.19, H 9.98, N 4. 32%.

For the compound **8a** [29], the general procedure was used with 20 g of choline, 49.35 g of decanoic acid, 23.23 mL of methane sulfonic acid, 35.06 g of sodium perchlorate and 9.7 g of potassium lactate.


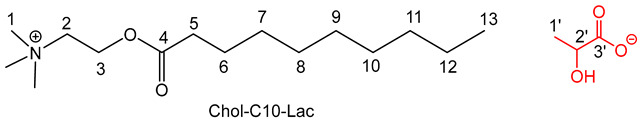

^1^HNMR: δ ppm (500 MHz; DMSO *d*_6_): 0.84 (t, 3H, *J* = 6.8 Hz, H13), 1.08 (d, 3H, *J* = 6.8 Hz, H1′); 1.23 (s large, 12H, H 7,8,9,10,11,12); 1.51–1.53 (m, 2H, H6), 2.32 (t, 2H, *J* = 6.8 Hz, H5); 3.17 (s, 9H, H1), 3.52 (q, 1H, *J* = 6.2 Hz, H2′), 3.72–3.74 (m, 2H, H2); 3.87 (s, 1H, OH); 4.44 (s, 2H, H3). ^13^C NMR: δ ppm (62.5 MHz; DMSO *d*_6_): 14.3 (C13), 22.0 (C1′), 22.6, 24.6, 29.3 (C7,8,9,10,11,12), 31.8 (C6), 33.9 (C5), 53.9 (C1), 58.2 (C2), 64.1 (C3), 67.5 (C2′), 172.8 (C4), 177.7 (C3′). IR: ν (cm^−1^) 1116 (C-O-H _Lactate_), 1595 (C=O_Lactate_), 1737 (C=O_ester_), 2912 (N-CH), 2812 (CH_3_-N). Anal. calcd.: C_18_H_37_NO_5_: C 62.22; H 10.73; N 4.03%; found: C 62.64; H 10.98; N 4. 41%.

For the compound **8b** [29], the general procedure was used with 20 g of choline, 49.35 g of decanoic acid, 23.23 mL of methane sulfonic acid, 35.06 g of sodium perchlorate and 12.51 g of potassium levulinate.


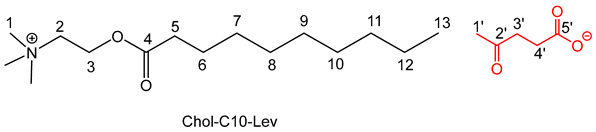

^1^HNMR: δ ppm (500 MHz; DMSO *d*_6_): 0.87 (t, 3H, *J* = 7.5 Hz, H13), 1.22 (s large, 12H, H 7,8,9,10,11,12), 1.51–1.53 (m, 2H, H6), 2.05 (s, 3H, H1′), 2.24 (t, 2H, *J* = 6.8 Hz, H5), 2.37 (t, 2 H, t, *J* = 7.5 Hz, H3′), 2.56 (t, 2 H, *J* = 7.5 Hz, H4′), 3.27 (s, 9H, H1), 3.72–3.74 (m, 2H, H2), 3.87 (s, 1H, OH), 4.53 (s, 2H, H3).^13^C NMR: δ ppm (62.5 MHz; DMSO *d*_6_): 14.2 (C13); 22.5 (C1′); 24.3, 28.5, 29.1, 29.2, 29.3, (C 7,8,9,10,11,12), 31.78 (C6); 32.7 (C5), 40.3 (C3′, C4′), 53.2 (C1); 58.4 (C2), 63.3 (C3), 172.8 (C4), 176.3 (C5′), 208.1 (C2′). IR: ν (cm^−1^) 1590 (COO^−^), 1715 (C=O_ketone_), 1746 (C=O_ester_), 2910 (N-CH), 2815 (CH_3_-N). Anal. calcd.: C_20_H_39_NO_5_: C 64.31; H 10.52; N 3.75%; found: C 64.19; H 10.08; N 3.32%.

For the compound **6**, the general procedure was used with 20 g of choline, 57.37 g of dodecanoic acid, 23.23 mL of methane sulfonic acid and 35.06 g of sodium perchlorate. A white powder was obtained with a yield of 75%.


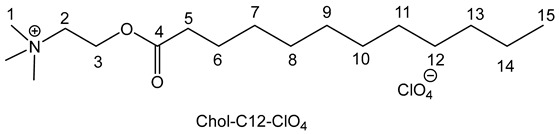

^1^HNMR: δ ppm (500 MHz; DMSO *d*_6_): 0.86 (t, 3H, *J* = 6.8 Hz, H15), 1.22 (s large, 16H, H 7,8,9,10,11,12,13,14), 1.53–1.56 (m, 2H, H6), 2.34 (t, 2H, *J* = 6.8 Hz, H5), 3.13 (s, 9H, H1), 3.64–3.67 (m, 2H, H2), 4.44 (s, 2H, H3). ^13^C NMR: δ ppm (62.5 MHz; DMSO *d*_6_): 14.4 (C15), 24.7, 28.5, 29.1, 29.2, 29.3, 29.5 (C 7,8,9,10,11,12, 13, 14), 31.9 (C6); 33.8 (C5), 53.4 (C1); 58.1 (C2), 64.3 (C3), 172.8 (C4). IR: ν (cm^−1^) 1062 and 616 (ClO_4_), 1733 (C=O), 2912 (N-CH), 2812 (CH_3_-N). Anal. calcd.: C_17_H_36_ClNO_6_: C 52.91, H 9.40, N 3.63%; found: C 52.76, H 9.13, N 3.36%.

For the compound **9a,** the general procedure was used with 20 g of compound **6**, and 11.07 g of potassium lactate. The liquid obtained in quantitative yield was white and highly viscous, tending to solidify at room temperature.


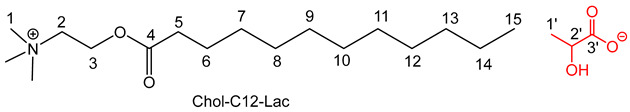

^1^HNMR: δ ppm (500 MHz; DMSO *d*_6_): 0.88 (t, 3H, *J* = 6.8 Hz, H15), 1,05 (d, 3H, *J* = 6.8 Hz, H1′), 1.23 (large s, 16H, H 7,8,9,10,11,12,13,14), 1.52–1.54 (m, 2H, H6), 2.33 (t, 2H, *J* = 6.8 Hz, H5), 3.14 (s, 9H, H1), 3.44 (q, 1H, *J* = 6.2 Hz, H2′), 3.63–3.66 (m, 2H, H2), 4.44 (s, 2H, H3). ^13^C NMR: δ ppm (62.5 MHz; DMSO *d*_6_): 14.4 (C15), 22.1 (C1′), 22.6, 24.7, 24.6, 29.3 (C7,8,9,10,11,12,13,14), 31.8 (C6), 33.8 (C5), 53.3 (C1), 58.2 (C2), 64.2 (C3), 172.4 (C4), 177.1 (C3′). IR: ν (cm^−1^) 1118 (C-O-H_Lactate_), 1595 (C=O_Lactate_), 1737 (C=O_ester_), 2912 (N-CH), 2812 (CH_3_-N). Anal. calcd.: C_20_H_41_NO_5_: C 63.96, H 11.00, N 3.73%; found: C 63.58, H 10.89, N 3.41%.

For the compound **9b**, the general procedure was used with 10 g of compound **6**, and 4 g of potassium levulinate. The liquid obtained in quantitative yield is white and highly viscous at room temperature.


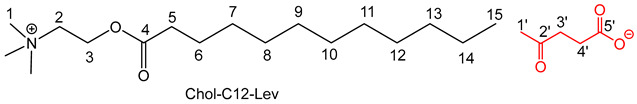

^1^HNMR: δ ppm (500 MHz; DMSO *d*_6_): 0.92 (t, 3H, *J* = 6.8 Hz, H15), 1.23 (large s, 16H, H 7,8,9,10,11,12,13,14), 1.51–1.54 (m, 2H, H6), 2.05 (s, 3H, H1′), 2.14 (t, 2H, *J* = 6.8 Hz, H5), 2.33 (t, 2 H, t, *J* = 6.8 Hz, H3′), 2.47 (t, 2 H, *J* = 6.8 Hz, H4′), 3.18 (s, 9H, H1), 3.72–3.74 (m, 2H, H2), 4.44 (s, 2H, H3).^13^C NMR: δ ppm (62.5 MHz; DMSO *d*_6_): 14.5 (C15); 22.4 (C1′), 24.5, 28.5, 29.1, 29.2, 29.3, 29.5 (C 7,8,9,10,11,12,13,15), 31.8 (C6), 34.0 (C5), 40.3 (C3′, C4′), 53.5 (C1), 58.2 (C2), 64.0 (C3), 172.7 (C4), 177.9 (C5′), 200.1 (C2′). IR: ν (cm^−1^) 1590 (COO^−^), 1715 (C=O_ketone_), 1746 (C=O_ester_), 2910 (N-CH), 2815 (CH_3_-N). Anal. calcd.: C_22_H_43_NO_5_: C 65.8, H 10.79, N 3.49%; found: C 65.59, H 10.38, N 3.22%.

### 2.4. Extraction of Curcuminoids

To extract the curcuminoids, 5 g of **7a** was heated to 70 °C in 1.5 mL of absolute ethanol, 1 g of turmeric powder was added, and the resulting mixture was heated to 60 °C for 30 min. After cooling the medium, 80 mL of distilled water was added, and the undissolved turmeric powder residues were filtered. A liquid–liquid extraction of the filtrate was performed with ethyl acetate (100 mL) to recover the curcuminoids in the organic phase. After evaporating the ethyl acetate, 100 mL of diethyl ether was added to precipitate the residual IL. To recover the maximum amount of curcuminoids, the precipitate obtained after adding ether was solubilized again in water (100 mL) and re-extracted with ether (100 mL). The two ether fractions were then combined, the ether evaporated under reduced pressure, and the resulting powder dried under vacuum. The curcuminoids were analyzed by TLC and UV. The aqueous phases were collected, and after evaporation of the water, the structurally unmodified IL (checked by NMR) could be reused for further extraction.

### 2.5. Extraction of Carvacrol

Mashed oregano leaves (1 g) were extracted at 60 °C for 30 min with a solution containing 5 g **7a** and 1.5 mL absolute ethanol, previously heated to 70 °C. Then was added water (80 mL) and, after filtration of the non-soluble residues as described above with curcuminoids, a liquid–liquid extraction of water–AcOEt (80/100) of the filtrate was carried out. After evaporation of the ethyl acetate, any IL still present was removed by washing with diethyl ether (4 × 100 mL). The ether phases were collected, dried, and evaporated under reduced pressure to furnish the carvacrol. As for the extraction of curcuminoids, the IL could be reused for other extractions batches (five cycles without loss of activity).


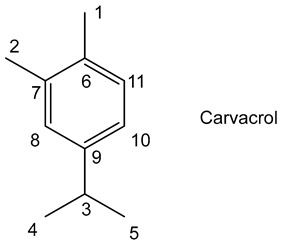

^1^HNMR: δ ppm (500 MHz; DMSO *d*_6_): 1.18 (d, 6H, *J* = 6.8 Hz, H4,H5), 2,13 (s, 3H, H1), 2.76–2.78 ppm (m, 1H, H3), 6.57 (d, 1H, *J* = 6.8 Hz, H11), 6.70 (s, 1H, H8), 6.95 (d, 1H, *J* = 6.8 Hz, H10), 9.04 (s, 1H, OH). ^13^C NMR: δ ppm (62.5 MHz; DMSO *d*_6_): 15.9 (C1), 24.7 (C4, C5), 33.6 (C3), 112.8 (C8), 117.1 (C10), 121.7 (C6), 131.0 (C11), 147.1 (C9), 155.7 (C7). IR: ν (cm^−1^) 3388 (Ar-O-H), 2957 (Csp^3^-H), 1584 (C=C Ar), 1249 (C-OH).

### 2.6. Cell Culture

MiaPaCa-2 pancreatic adenocarcinoma cells were obtained from the American Type Culture Collection (ATCC). They were grown in Dulbecco’s modified Eagle’s medium (DMEM) containing 10% fetal bovine serum (FBS) and 1 IU/mL penicillin/streptomycin.

### 2.7. Cytotoxicity Evaluation

Two different methods were used: (i) reduction of a tetrazolium salt (WST1) by mitochondrial oxido-reductases [54] and (ii) staining of cells with crystal violet, a triarylmethane dye that binds cell DNA and protein [55].

Cells were seeded in 96-well plates (10,000 cells/well) in DMEM with 10% FBS and allowed to settle for 24 h. The medium was removed, and effectors were added to the cells at various concentrations in DMEM without FBS for 48 h.

WST1 assay: At the end of the incubation period, the medium was replaced by fresh medium containing 10% WST-1 reagent, and cells were incubated for 30 min at 37 °C. The absorbance was measured at 450 nm using a microplate spectrophotometer (SPECTROstar^®^ Nano, BMG Labtech, Champigny-sur-Marne, France).

Crystal violet staining: At the end of the incubation period, cells were washed with Phosphate Buffered Saline (PBS), fixed with 1.1% (*v*/*v*) glutaraldehyde in PBS for 20 min, and washed with PBS. They were then stained with 0.1% (*m*/*v*) crystal violet in HEPES (0.2 M, pH 6) for 20 min and washed with distilled water. Dye was eluted with 10% (*v*/*v*) acetic acid and absorbance was read at 560 nm.

### 2.8. Cell Growth Measurement

Cells were seeded in 96-well plates (10,000 cells/well) in DMEM with 10% FBS and allowed to settle for 24 h. The medium was then removed, cells were washed twice with PBS, and effectors were added to the cells at various concentrations.

7a-extracted curcuminoid powder was dissolved in DMSO to get a 10 mg/mL stock solution and then diluted in a culture medium with 10% FBS to final concentrations ranging from 0 to 50 µg/mL (0, 2.5, 5, 7.5, 10, 15, 20, 30, 40, 50 µg/mL). Commercial curcuminoid (Sigma–Aldrich, ref C1386; lot: MKCD2451) powder was dissolved in DMSO to get a 10 mg/mL stock solution and then diluted in a culture medium with 10% FBS to final concentrations ranging from 0 to 25 µg/mL (0, 0.63, 1.25, 2.5, 5, 7.5, 10, 15, 20, 25 µg/mL). The same amount of DMSO was added to the culture media for each condition, including control condition (medium without effectors referred as 0 µg/mL): 0.2% DMSO final concentration.

Cells were imaged every 4 h for 72 h using the phase contrast channel in an Incucyte^®^ S3 (Sartorius, Göttingen, Germany). Four sets of phase contrast images from distinct regions within each well were taken at the intervals indicated in the figures using a 10× objective. Incucyte^®^ S3 image analysis software v2024A was set to detect the edges of the cells and to determine their confluence in percentage. Graphs were generated with the Incucyte^®^ image analysis software v2024A graph/export functions and Microsoft Excel software.

### 2.9. Scratch Assay

Cells were seeded in Incucyte^®^ Imagelock 96-well microplate (40,000 cells/well) in DMEM with 10% FBS and allowed to settle for 24 h. Scratches were performed using the WoundMaker™ tool (Sartorius, Göttingen, Germany). The medium was then removed. Cells were washed twice with PBS and effectors were added to the cells at various concentrations in DMEM with 10% FBS.

The 7a-extracted curcuminoid powder was dissolved in DMSO to get a 10 mg/mL stock solution and then diluted in a culture medium with 10% FBS to 0, 20 and 30 µg/mL final concentrations. Commercial curcuminoid (Sigma–Aldrich, ref C1386; lot: MKCD2451) powder was dissolved in DMSO to get a 10 mg/mL stock solution and then diluted in a culture medium with 10% FBS to 0, 1.25, 2.5 µg/mL final concentrations. The same amount of DMSO was added to the culture media for each condition, including the control condition (medium without effectors, referred to as 0 µg/mL): 0.2% DMSO final concentration.

Wound closure followed using the Incucyte^®^ S3 Live-Cell Analysis System according to the manufacturer’s instructions. Every 4 h for 96 h, pictures of each well were taken up. Cell migration rates are reported as the relative wound density calculated using an algorithm measuring cell density in the wound area relative to the cell density outside of the wound area. Graphs were generated with the Incucyte^®^ image analysis software v2024A graph/export functions and Microsoft Excel software.

## 3. Results and Discussion

**Scheme 1:**
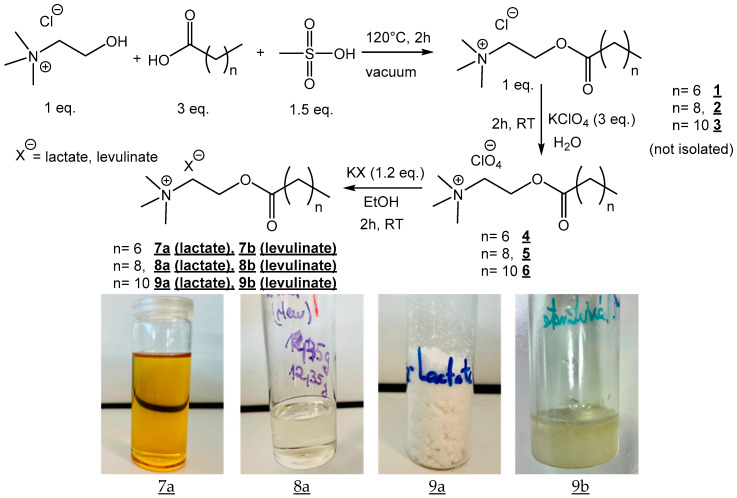
Synthesis of cholinium lactate and levulinate.

The preparation of the choline-based IL involved a three-step procedure (esterification followed by two anionic metathesis) with good yields (70–85%) according procedures previously described [15a,c] for compounds **4**, **5**, **7a**, **7b**, **8a** and **8b** (Scheme 1). Concerning compounds **6**, **9a** and **9b**, the same methodology was employed, and compounds **9a** and **9b** were obtained with 75 and 80% yields, respectively (Table 1).

The first extraction tests were carried out with curcuma longa (1 g) and oregano (1 g). Three synthesized ILs **7a**, **8a** and **9a** were used for the extraction of these two plants for comparison with the best classical extraction methods for these plants described in the literature [36,38,39], according to experimental procedure. Compound **9a** was the IL that extracted the most active ingredients from both *Curcuma longa* L. and oregano (Table 2). This greater efficiency can be explained by the influence of the carbon chain length (12 carbon atoms) being the least polar. Unlike other anions, which can be neutral or hydrophobic, lactate provides the IL a hydrophilic property, with the possibility of establishing hydrogen- or ionic-type interactions that play an important role in biomolecule extraction. It should be noted that **9a** presents an extracting efficiency for these two plants comparable to conventional methods, and that it can be reused at least four times without loss of extraction activity, which is not the case with conventional organic solvents.

Residual compound **9a** is present in the extracts of curcuminoids, which was confirmed by gas chromatography (GC-MS) and NMR. As curcuminoids have a broad absorption spectrum, with a maximum around 425 nm, UV-Visible spectroscopy at 425 nm enabled us to estimate, according to the calibration below, a quantity of 0.21 g of curcuminoids per gram of turmeric powder; for 1 g of turmeric powder, the extraction yield was therefore 21%, or 95% of the total curcuminoids contained in curcuma longa. The extract was then purified with cold diethyl ether, as this solvent solubilizes curcuminoids well and precipitates residual IL.

The oregano extract obtained with **7a**, **8a** and **9a** contained only carvacrol. This result was confirmed by TLC, ^1^H NMR and GC/MS. The best carvacrol extraction yields were obtained with **9a**, which extracted 29% carvacrol with traces of IL. After optimization, we obtained 27% carvacrol by mass per gram of oregano leaves, i.e., 69% of the total carvacrol contained in oregano. This yield was determined using a calibration established at 275 nm (carvacrol’s absorption wavelength). Extraction of oregano with synthesized ILs is more selective than with conventional solvents because ethanol extracts contain not only carvacrol but also other compounds such as thymol, linalool and terpenes. This selectivity can be explained by the strong non-covalent interaction between the lactate anion of the IL and carvacrol.

After extraction, IL **9a** was able to be recovered by evaporating the water, then reused for subsequent extraction. The recycling capacity of **9a** was demonstrated in the same conditions of extraction as the curcuminoids (21, 20, 21 and 19%, respectively, for each run) and the carvacrol (27, 26, 27 and 24%, respectively, for each run).

### 3.1. Analysis of IL Cytotoxicity on MiaPaca-2 Cells

**Figure 2:**
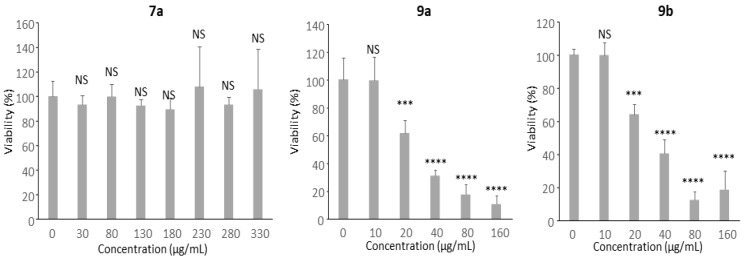
Cytotoxicity of **7a**, **9a** and **9b** ILs on MiaPaca-2 cells evaluated using the WST-1 assay. Results are expressed as means ± SD. Statistical significance was studied using *t*-tests. NS: not significant; ***: *p* < 0.001; ****: *p* < 0.0001. *n* = 7.

**Figure 3:**
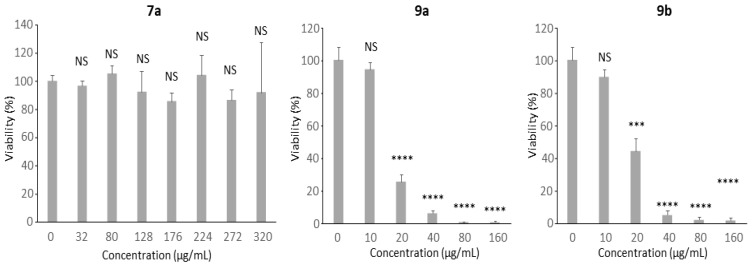
Cytotoxicity of **7a**, **9a** and **9b** ILs on MiaPaca-2 cells evaluated using crystal violet staining. Results are expressed as means ± SD. Statistical significance was studied using *t*-test. NS: not significant; ***: *p* < 0.001; ****: *p* < 0.0001. *n* = 7.

MiaPaca-2 cells were incubated for 48 h in the presence of increasing concentrations of each IL. Using the WST-1 assay (Figure 2), for the different concentrations tested, Chol-C8-lactate **7a** did not appear to be toxic to cells, while Chol-C12-lactate **9a** and Chol-C12-levulinate **9b** showed similar toxicity from 20 µg/mL, with IC50 of 22.2 and 24.1 µg/mL, respectively. Very similar results were obtained with the crystal violet assay (Figure 3).

The ILs containing 12 carbon atoms **9** appear to be more cytotoxic than those containing eight carbon atoms **7**. This observation is fully in accordance with the work of Arakelyan et al. [56], where it was previously reported that the length of the alkyl side chain in the cation ILs was a major attribute related to the toxicity. The nature of the anion is also important, and we observed that **9b** is slightly lower than **9a**. This difference between lactate and levulinate ILs has been also observed in previous works [14].

### 3.2. Effect of Curcuminoids on Cell Biological Activity

Solvent **7a** was chosen to extract bioactive compounds, as it does not induce a cytotoxic effect on its own.

As a proof of concept, **7a**-extracted curcuminoid biological activity was compared to commercially available curcuminoids (Sigma–Aldrich, ref C1386; lot: MKCD2451).

**Figure 4:**
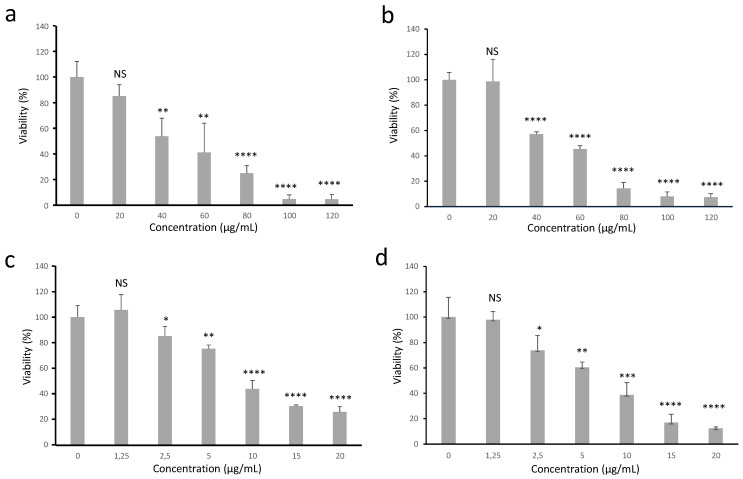
MiaPaca-2 cell toxicity of curcuminoid extract analyzed using WST-1 (**a**) and crystal violet (**b**) assay compared to commercial curcuminoid analyzed using WST-1 (**c**) and crystal violet (**d**), Results are expressed as means +/− SD. Statistical significance was studied using *t*-tests. NS: not significant; *: *p* < 0.05; **: *p* < 0.01; ***: *p* < 0.001; ****: *p* < 0.0001. *n* = 7; N = 2.

As shown in the WST-1 assay, MiaPaca-2 cell viability decreased dose-dependently following 48 h incubation with IL-extracted curcuminoids (IC50 = 50 µg/mL) (Figure 4a). The results were compared to those obtained with commercial curcuminoids. Toxic effects appeared at 2.5 µg/mL concentration and cell viability, then decreased dose-dependently (IC50 = 8.5 µg/mL) (Figure 4c). The results corroborate those obtained using crystal violet assay (Figure 4b,d).

**Figure 5:**
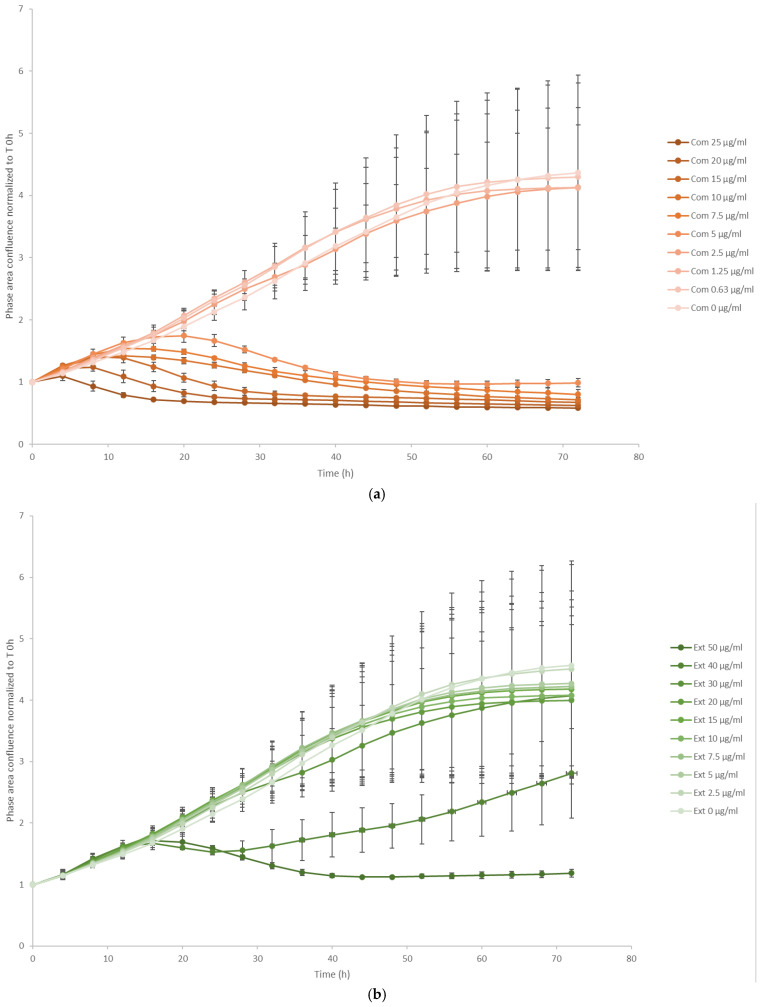
MiaPaca-2 cell growth analyzed using the Incucyte S3 v2024A software. (**a**) Liquid ionic extracted curcuminoids. (**b**) Commercial curcuminoids. Results are expressed as means ± SD. *n* = 3; N = 3.

MiaPaca-2 cell growth was assessed using the Incucyte^®^ S3 imaging system after 48 h of incubation with increasing amounts of curcuminoids (Figure 5). Growth of cells incubated with 2.5 to 30 µg/mL of IL-extracted curcuminoids was quite similar to the control condition. It slowed down in the presence of 40 µg/mL of extract and was nullified in the presence of 50 µg/mL extract. After 48 h of incubation, an EC50 of 38.4 ± 0.6 µg/mL was calculated using the Incucyte^®^ Software. By comparison, growth of cells cultured with 0.63 to 2.5 µg/mL of commercial curcuminoids was similar to the control condition. There was none for the above concentrations. A 3.6 ± 0.3 µg/mL EC50 was determined after a 48 h-incubation period with this compound.

**Figure 6:**
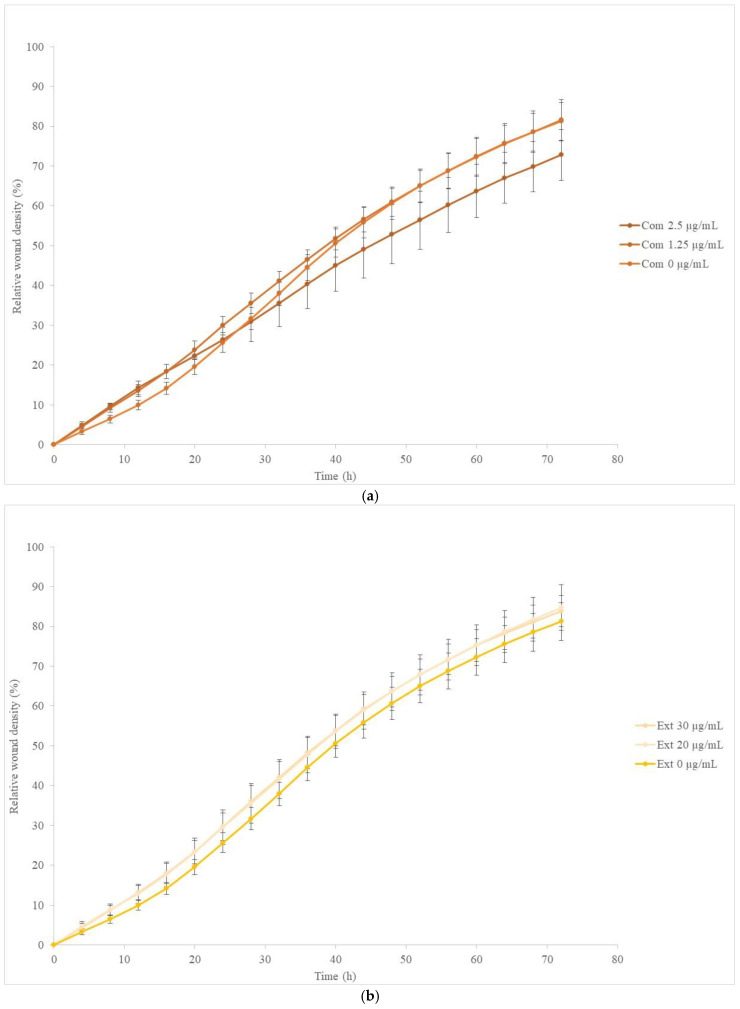
MiaPaca-2 cell migration analyzed using Incucyte Scratch wound analysis software v2024A. (**a**) Liquid ionic extracted curcuminoids. (**b**) Commercial curcuminoids. Results are expressed as means ± SD. *n* = 6; N = 3.

The MiaPaca-2 cell migration was also studied using the Incucyte^®^ Scratch Wound Analysis Software Module. No differences were observed in the curcuminoid extracts at the different concentrations tested (Figure 6).

Previous work from Bemonte et al. [57] reported a 10-fold increase in MiaPaca-2 cell apoptosis following 48 h incubation with 50 µM curcuminoids, corresponding to approximatively 20 µg/mL of curcuminoids. These results are in agreement with ours. Indeed, at the same concentration, we observed a large decrease in cell viability. An IC50 of 11.16 µM (4 µg/mL) was observed by Friedman et al. [58] in the cytotoxicity assay performed on MiaPaca-2 after 72 h of incubation with curcuminoids. After 24 h of incubation with 80 µM (30 µM) curcuminoids, Yang et al. [59] reported a 60% decrease in MiaPac-2 cell viability and 50% in Panc-1. In the same way, an IC50 of 25 µM (9.2 µM) was reported by Lev-Ari et al. [36] on pancreatic PANC-1 cell cytotoxicity. Liu et al. [46] previously reported IC50 of 9.87 and 13.49 µM, respectively, in cytotoxic assays on Panc-1 and MiaPaca-2 cells. All these results are consistent with ours, even though IC50 values may vary according to experimental conditions (incubation time, cytotoxicity test used, cell type). The results obtained in cell growth experiments confirm those obtained in the cytotoxic assay. At non-cytototoxic doses, we did not see any significant effect of curcuminoids on MiaPaca-2 cell migration. Liu et al. [60] performed migration assays in a scratch wound model and showed an inhibition of PANC-1 cell migration after a 24 h incubation period with 5 µM (1.8 µg/mL) curcuminoids. However, no results were presented for MiaPaca-2 cell migration, even though both cell types were studied for cytotoxic effects as reported above. In our study, at the 2.5 µg/mL concentration, we did not see any difference from the control condition regarding MiaPaca-2 cell migration.

Concerning the curcuminoids extracted with IL **7**, we observed a six-fold less cytotoxic effect than the one observed with the commercial curcuminoids. This may be due either to a better solubility of curcuminoids in the ionic medium, implying an interaction between curcuminoids and the IL that slows down the curcuminoid–bioreceptor interaction [61], or to the composition of the extract in terms of curcumins I, II and III (Figure 1) in the ionic extract, which is not the same as that of commercial curcumins. HPLC and UV assay studies are scheduled in this field.

## 4. Conclusions

In the present work, six choline ester-based ILs were synthetized from saturated fatty acids (octanoic, decanoic and dodecanoic acids) and choline with conversions of 90, 75 and 80%, respectively. The ILs containing 12 carbon atoms have not been described in the literature and proved to be of interest for the extraction of curcuminoids or carvacrol from turmeric or oregano respectively, as the yields of extracted curcuminoids and carvacrol exceed the yields described in the literature for conventional organic solvents with or without activation methods. What is more, these solvents can be reused at least four times without loss of activity. The extraction of the carvacrol from oregano is also very selective. Furthermore, improvements in extracting yields through ultrasound activation are also in progress.

The cytotoxic activity of curcuminoids extracts containing traces of residual IL is six-fold weaker than that of commercial curcuminoids, requiring further work to be carried out in the near future to determine the ratios among the different curcumins. The cytotoxic activity of carvacrol or carvacrol extracts is also under investigation.

## Figures and Tables

**Table 1:** Cholinium-based esters with lactate and levulinate as counter anion.

Ionic Liquids	Aspect	Degradation Temperature (°C)	Viscosity (cP) at 60 °C
Chol-C8-Lac **7a** [14a]	Colorless oil	218	49.3
Chol-C8-Lev **7b** [14a]	Light beige wax	229	67.8
Chol-C10-Lac **8a** [14c]	Slightly yellow wax	226	nd
Chol-C10-Lev **8b** [14c]	Slightly yellow wax	232	nd
Chol-C12-Lac **9a**	White wax	248	nd
Chol-C12-Lev **9b**	White wax	247	nd

**Table 2:** Extractions of curcuma and oregano using **7a**, **8a** and **9a** as extracting solvents.

Starting Material	Extracting Solvent	Conditions	Bioactive Compounds	Extraction Yields ^a^
Curcuma (1 g)	**7a** (6 g)	2 h, 60 °C	Curcuminoids	15
Curcuma (1 g)	**8a** (6 g)	2 h, 60 °C	Curcuminoids	16
Curcuma (1 g)	**9a** (5 g)	+EtOH (1.5 mL), 2 h, 60 °C	Curcuminoids	21
Curcuma (0.6 g)	Acetone	Acetone (6 g), 2 h, 60 °C	Curcuminoids	16
Oregano (1 g)	**7a**	2 h, 60 °C	Carvacrol	12
Oregano (1 g)	**8a**	2 h, 60 °C	Carvacrol	18
Oregano (1 g)	**9a** (5 g)	+EtOH (1.5 mL), 2 h, 60 °C	Carvacrol	27
Oregano (0.5 g)	Absolute EtOH	EtOH (50 mL), 6 h, 95 °C	Carvacrol	18

^a^: gravimetry and UV determination.

## Data Availability

The data that support the findings of this study are available from the corresponding author.

## Supplementary Materials

The following supporting information can be downloaded at: https://www.mdpi.com/article/10.3390/molecules30051180/s1, Figure S1: ^1^H NMR spectrum of curcuminoids extracted with IL Chol-C12-Lact; Figure S2: ^1^H NMR spectrum of carvacrol extracted with IL Chol-C12-Lact; Figure S3: ^13^C NMR spectrum of carvacrol extracted with IL Chol-C12-Lact; Figure S4: IR spectrum of curcuminoids extracted with IL Chol-C12-Lact; Figure S5: IR spectrum of carvacrol extracted with IL Chol-C12-Lact; Figure S6: ^1^H NMR spectrum of the ester Chol-C8-ClO_4_; Figure S7: ^13^C NMR spectrum of the ester Chol-C8-ClO_4_; Figure S8: ^1^H NMR spectrum of the ester Chol-C10-ClO_4_; Figure S9: ^13^C NMR spectrum of the ester Chol-C10-ClO_4_; Figure S10: ^1^H NMR spectrum of the ester Chol-C12-ClO_4_; Figure S11: ^13^C NMR spectrum of the ester Chol-C12-ClO_4_; Figure S12: ^1^H NMR spectrum of IL Chol-C8-Lact; Figure S13: ^13^C NMR spectrum of the IL Chol-C8-Lact; Figure S14: ^1^H NMR spectrum of the IL Chol-C10-Lact; Figure S15: ^13^C NMR spectrum of the IL Chol-C10-Lact; Figure S16: ^1^H NMR spectrum of the IL Chol-C12-Lact; Figure S17: ^13^C NMR spectrum of the IL Chol-C12-Lact; Figure S18: ^1^H NMR spectrum of IL Chol-C12-Lev; Figure S19: ^13^C NMR spectrum of the IL Chol-C12-Lev; Figure S20: IR spectrum of the ester Chol-C12-ClO_4_; Figure S21: IR spectrum of the ILChol-C12-Lac; Figure S22: Thermo-gravimetric analysis (TGA) curves for ionic liquids; Figure S23: Viscosity of ionic liquids and TLC extracted curcumins.
