# Supplementary material for: Extraction of Curcuminoids and Carvacrol with Biobased Ionic Liquids—Evaluation of Anti-Cancer Properties of Curcuminoid Extracts
Source: Molecules. 2025 Mar 6;30(5):1180. doi: 10.3390/molecules30051180 (PMC11901620; doi:10.3390/molecules30051180)
Supplement: Supplementary file 1 [file molecules-30-01180-s001.zip › molecules-3481726-supplementary.pdf]

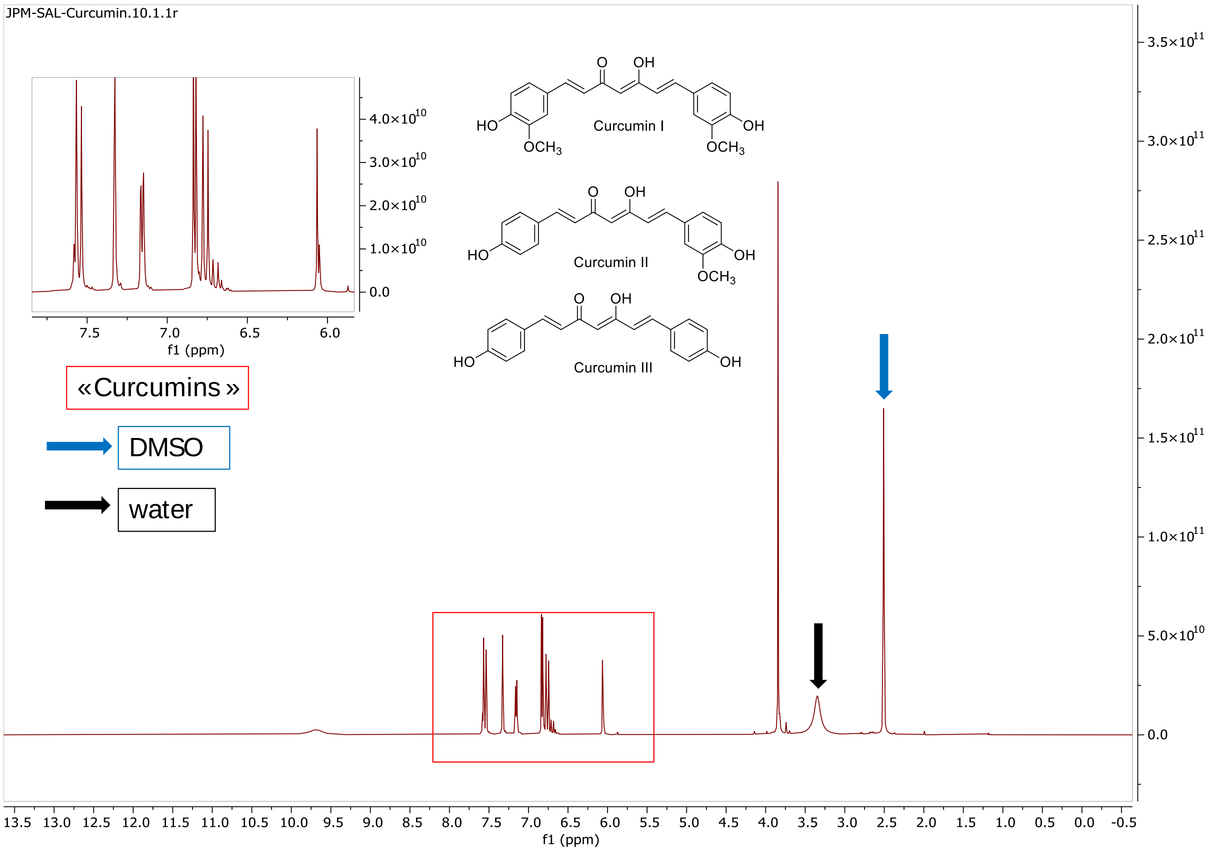

Figure S1: ^{1}H NMR spectrum of curcuminoids extracted with IL Chol-C12-Lact

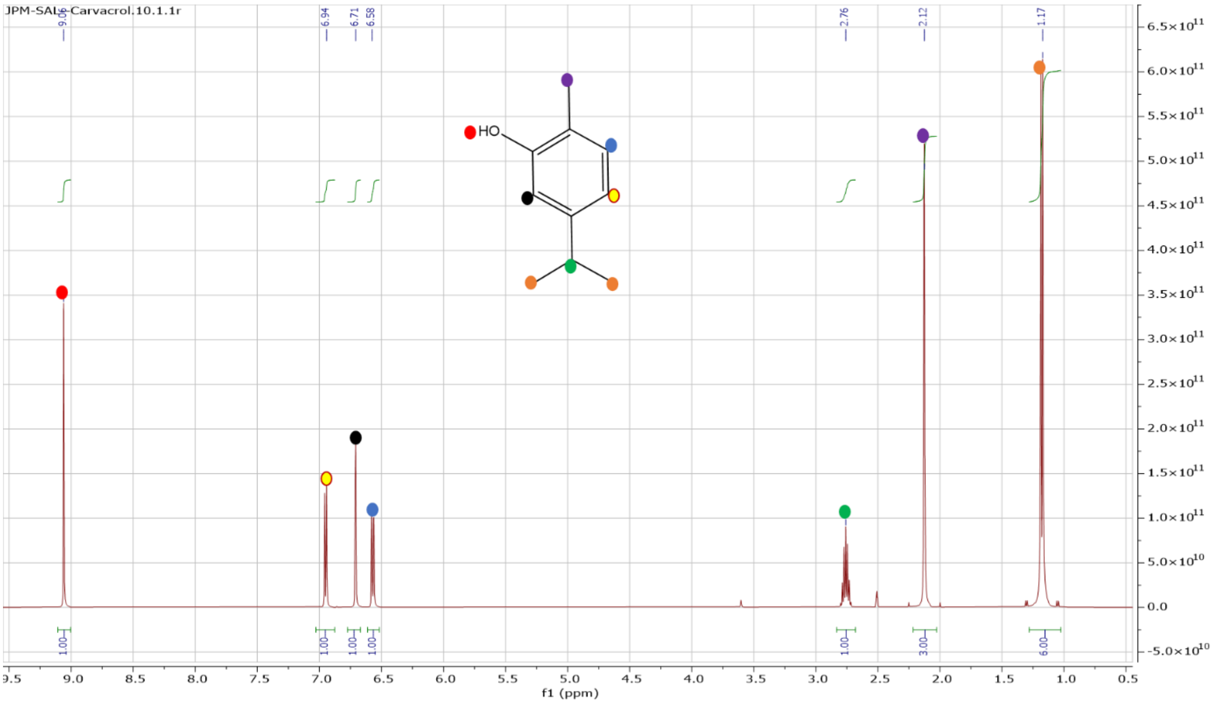

Figure S2: ^{1}H NMR spectrum of carvacrol extracted with IL Chol-C12-Lact

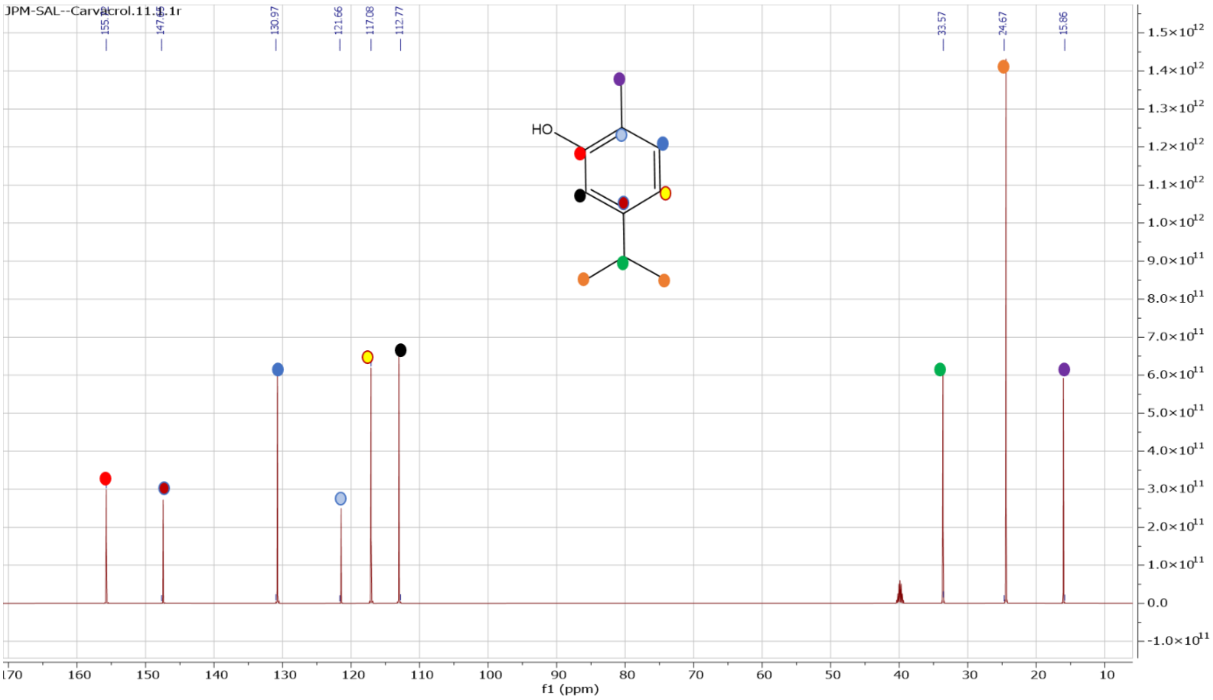

Figure S3: ^{13}C NMR spectrum of carvacrol extracted with IL Chol-C12-Lact

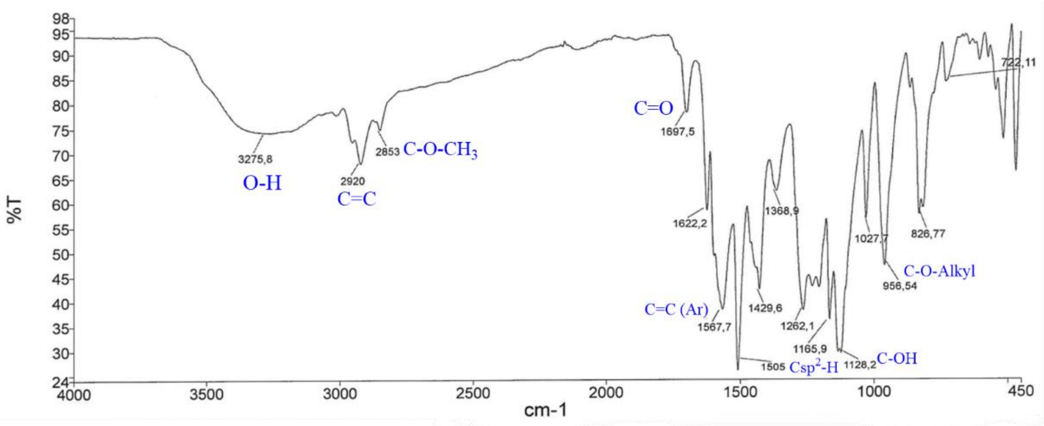

Figure S4: IR spectrum of curcuminoids extracted with IL Chol-C12-Lact

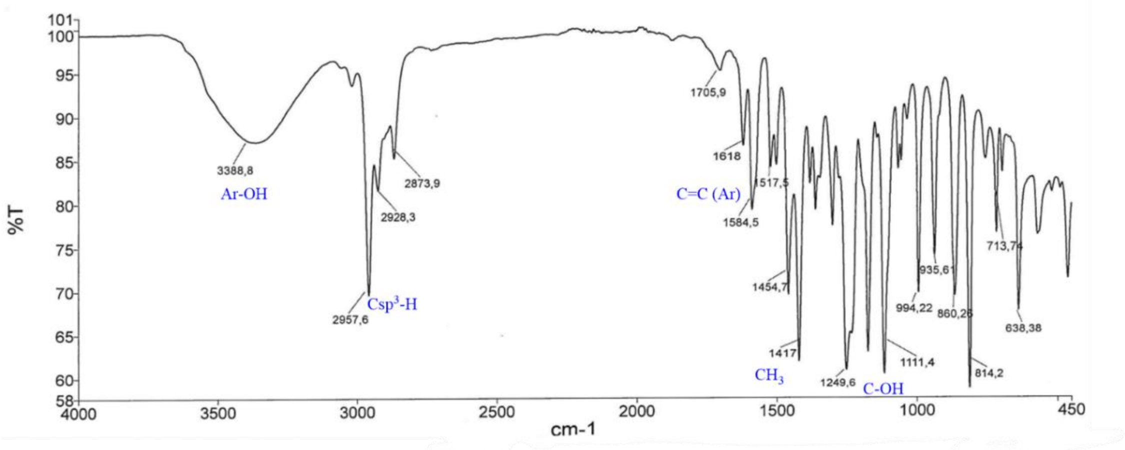

Figure S5: IR spectrum of carvacrol extracted with IL Chol-C12-Lact

Physical and chemical characterisation of ionic liquids

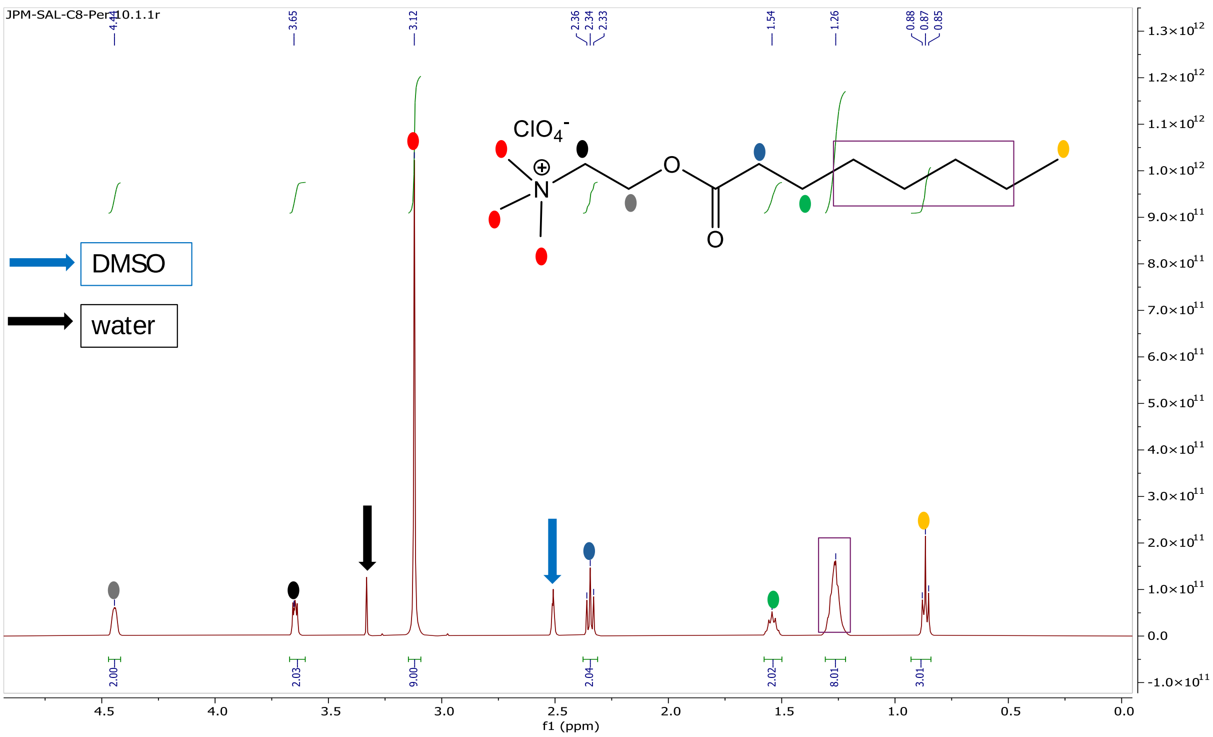

Figure S6: ^{1}H NMR spectrum of the ester Chol-C8-ClO_4

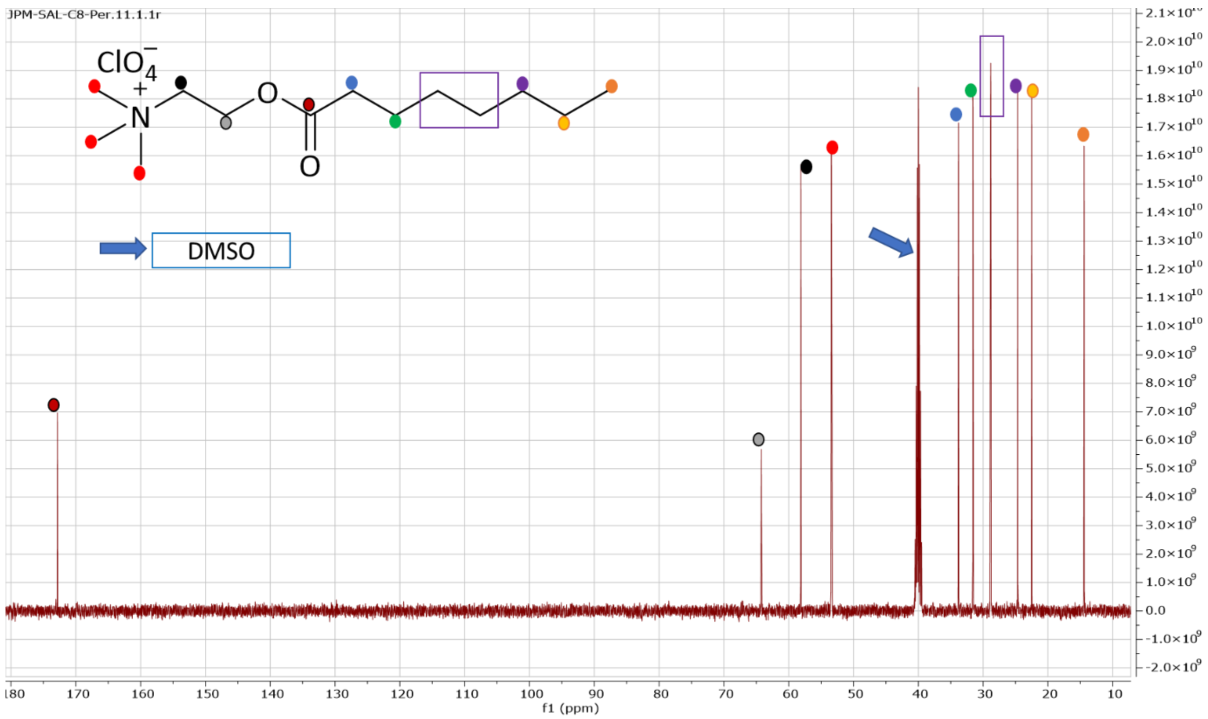

Figure S7: ^{13}C NMR spectrum of the ester Chol-C8-ClO_4

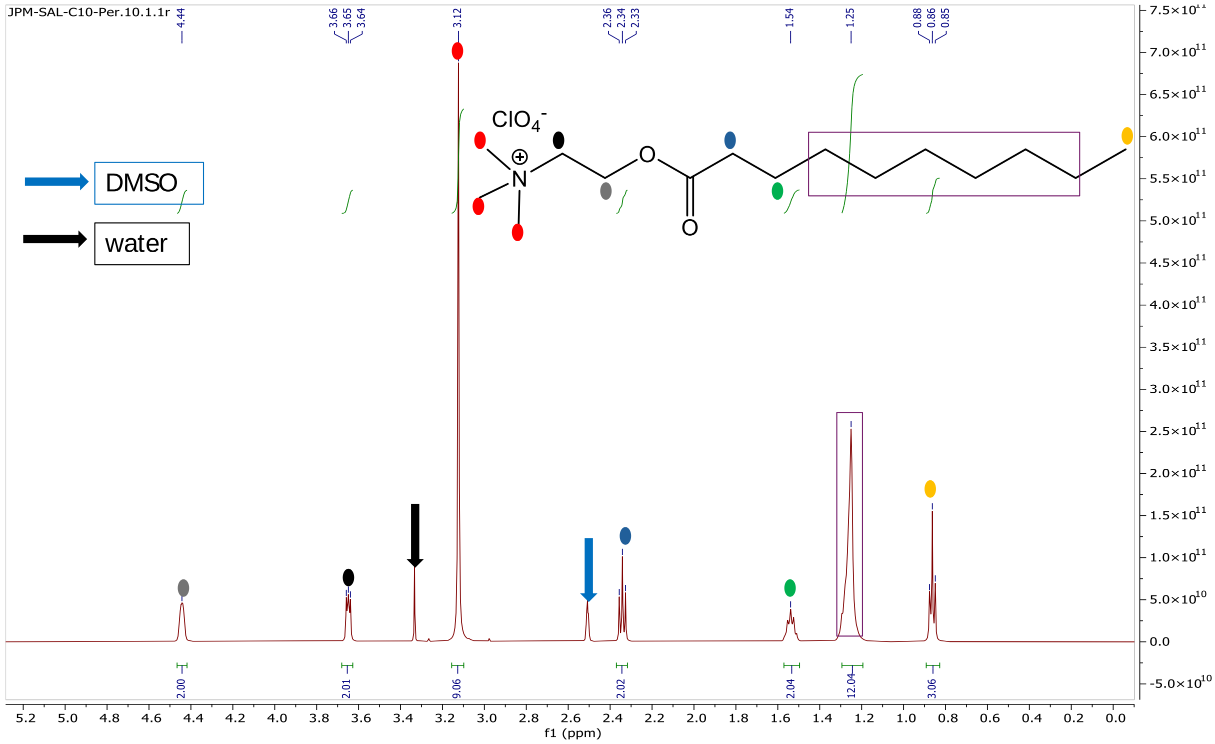

Figure S8: ^{1}H NMR spectrum of the ester Chol-C10-ClO_4

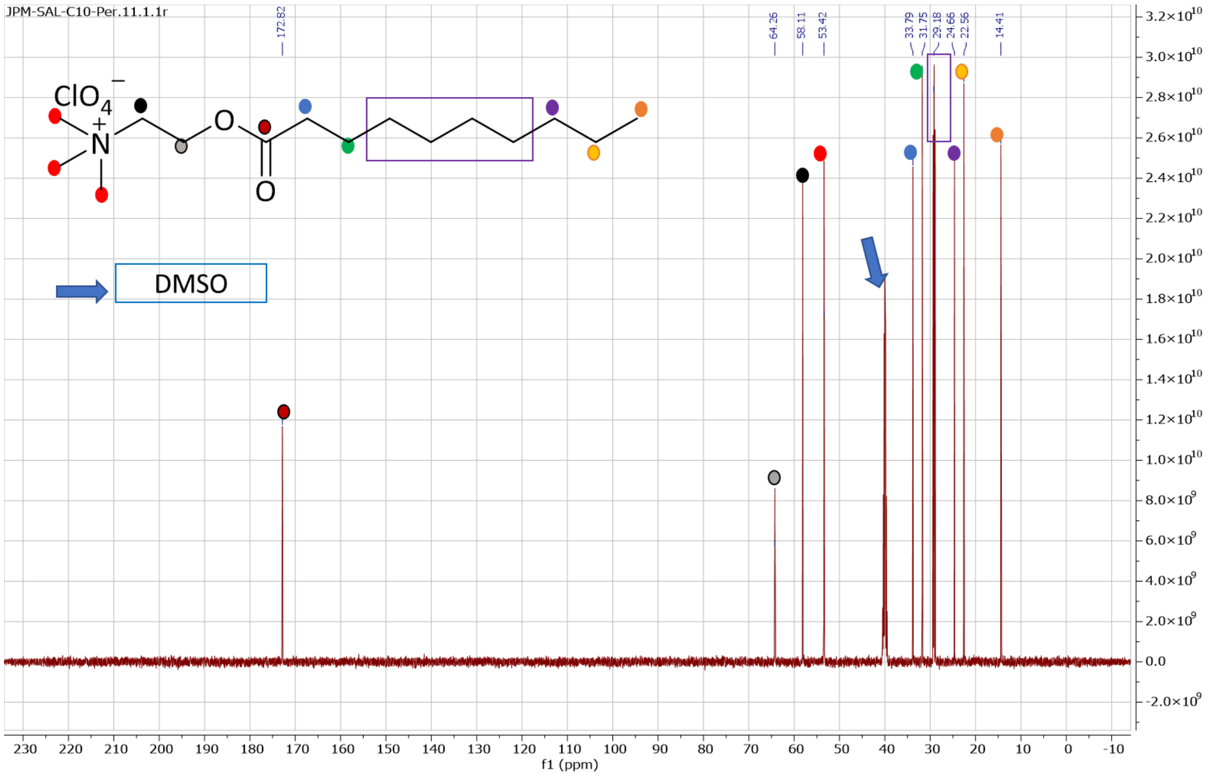

Figure S9: ^{13}C NMR spectrum of the ester Chol-C10-ClO_4

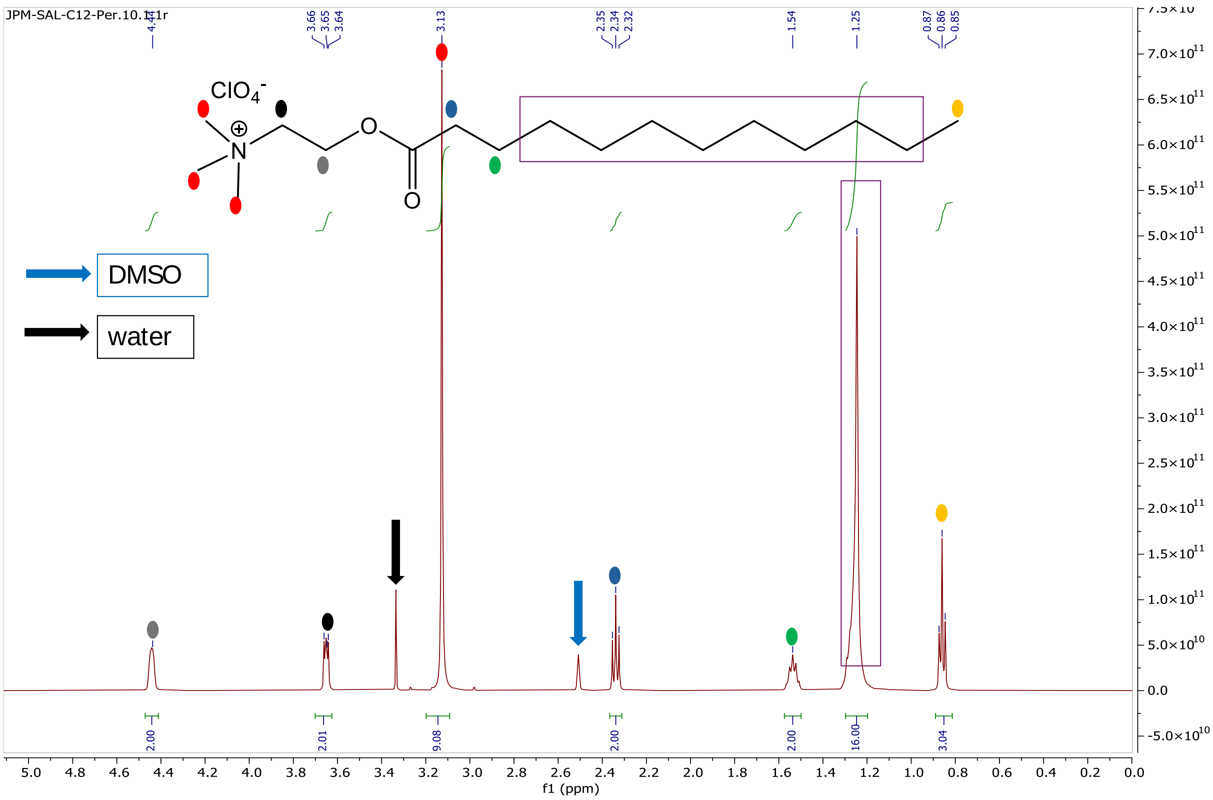

Figure S10: ^{1}H NMR spectrum of the ester Chol-C12-ClO_4

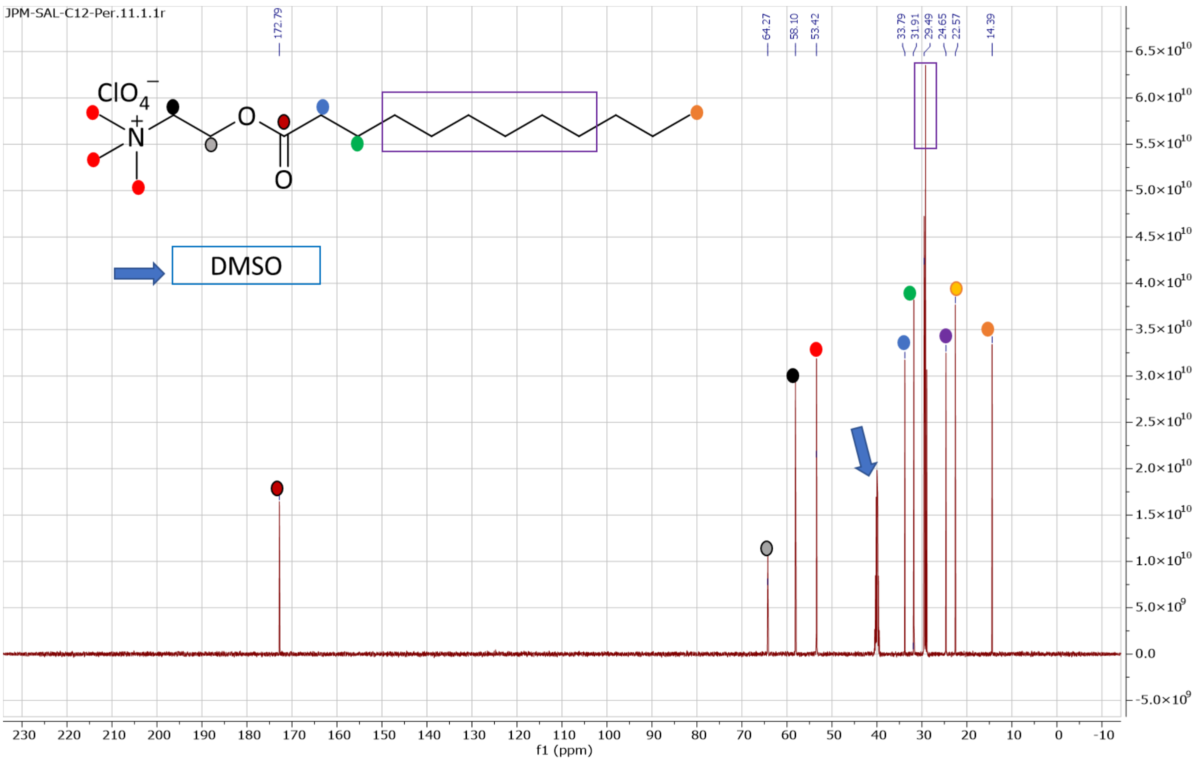

Figure S11: ^{13}C NMR spectrum of the ester Chol-C12-ClO_4

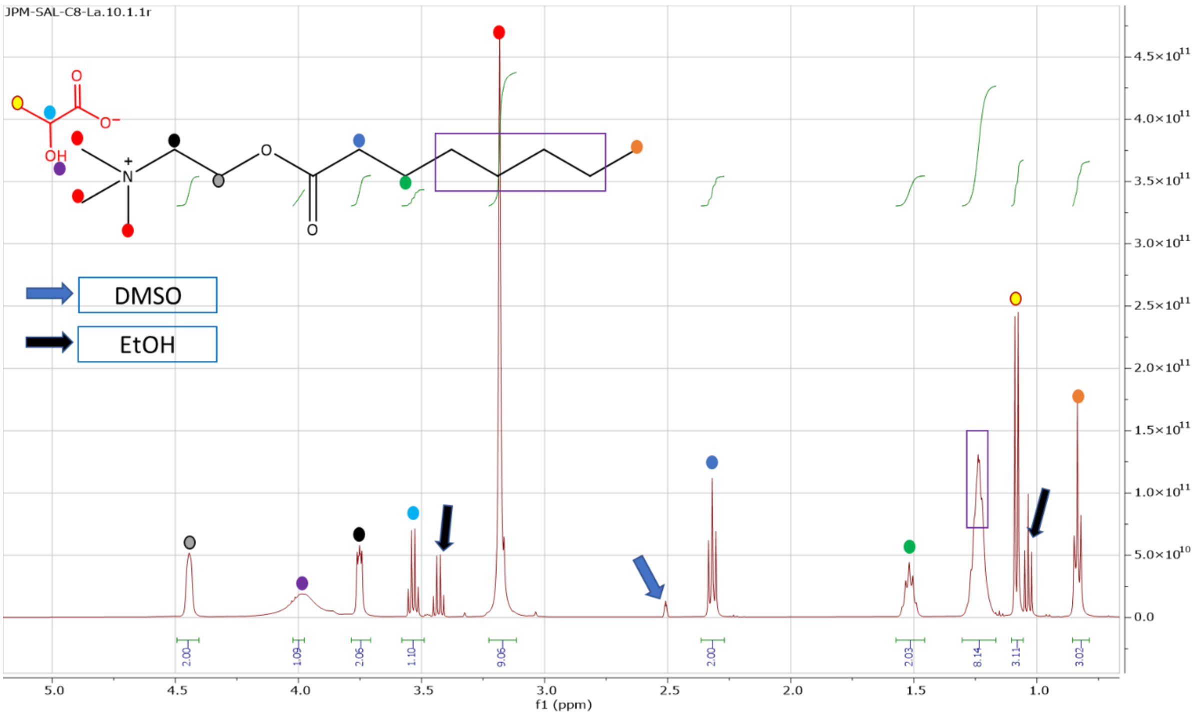

Figure S12: ^{1}H NMR spectrum of IL Chol-C8-Lact

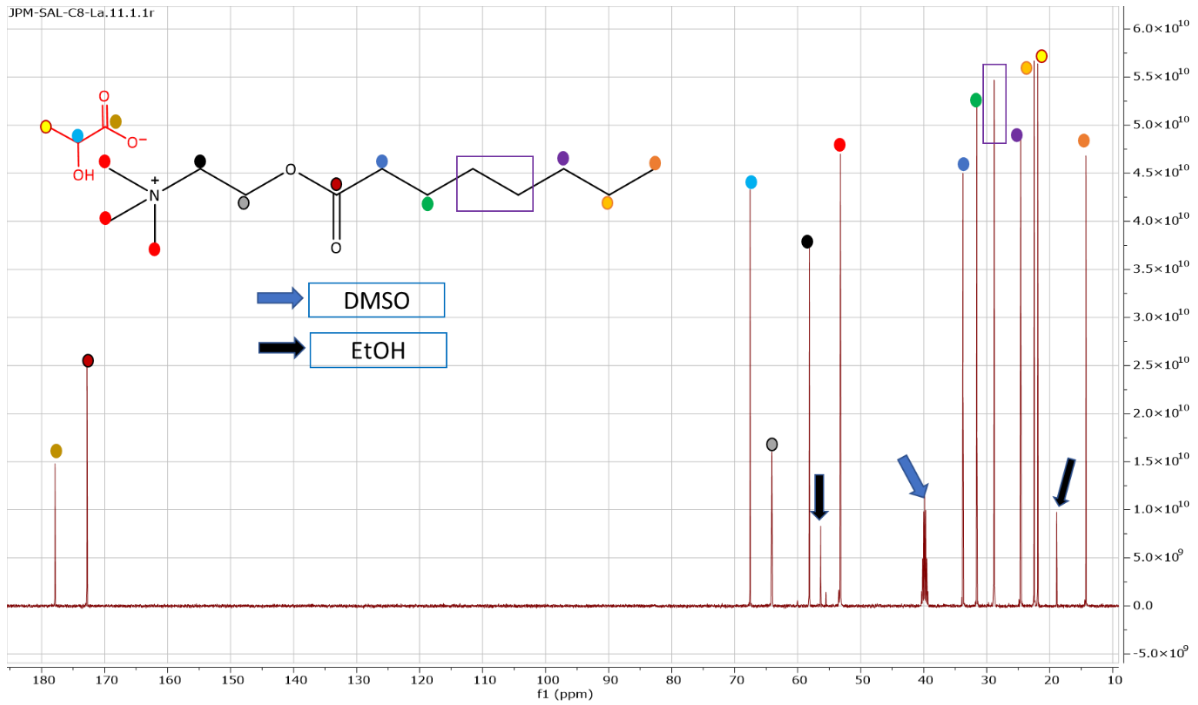

Figure S13: ^{13}C NMR spectrum of the IL Chol-C8-Lact

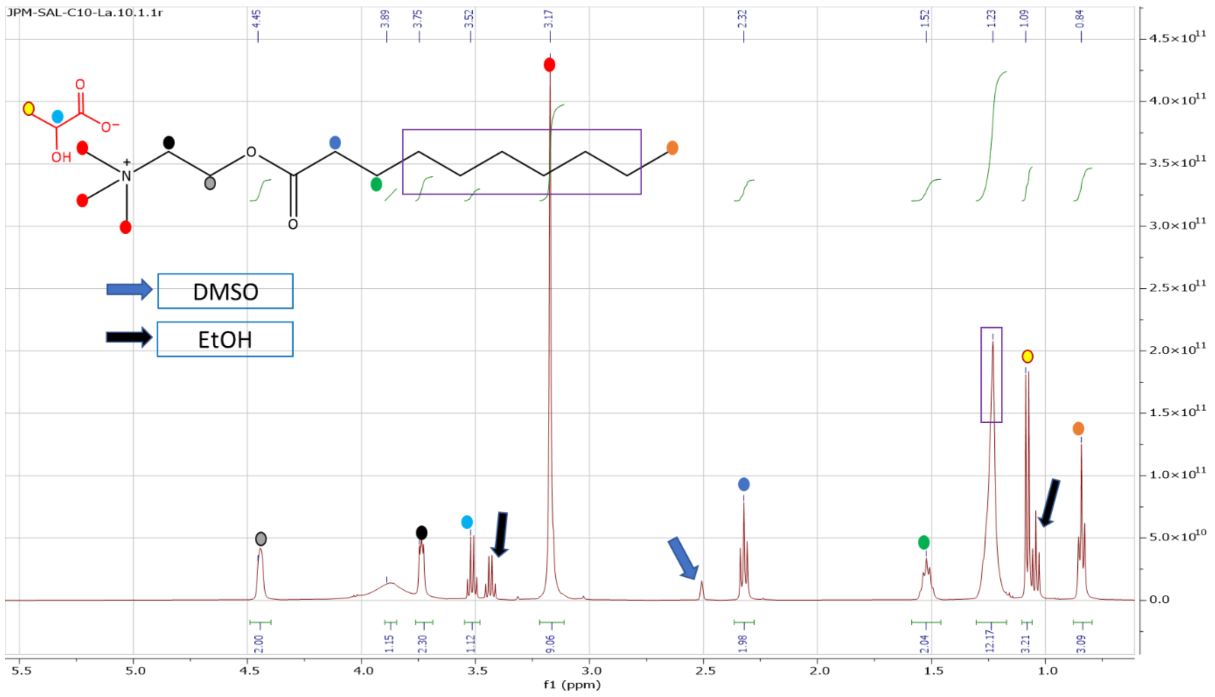

Figure S14: ^{1}H NMR spectrum of the IL Chol-C10-Lact

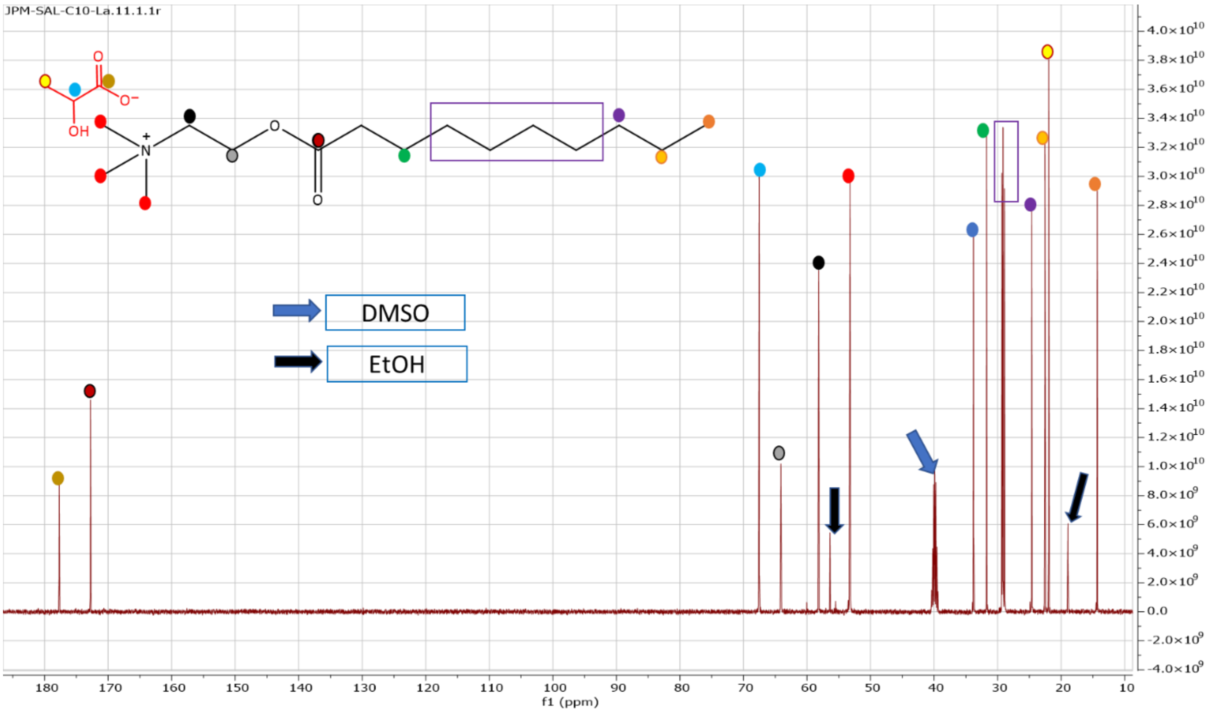

Figure S15: ^{13}C NMR spectrum of the IL Chol-C10-Lact

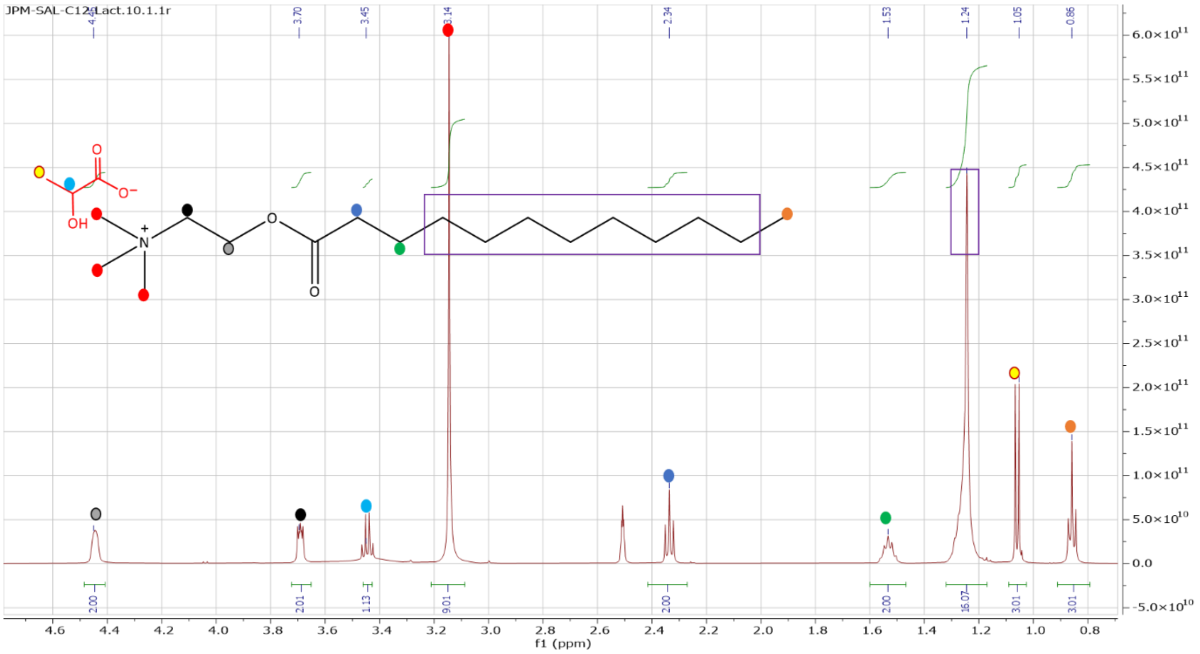

Figure S16: ^{1}H NMR spectrum of the IL Chol-C12-Lact

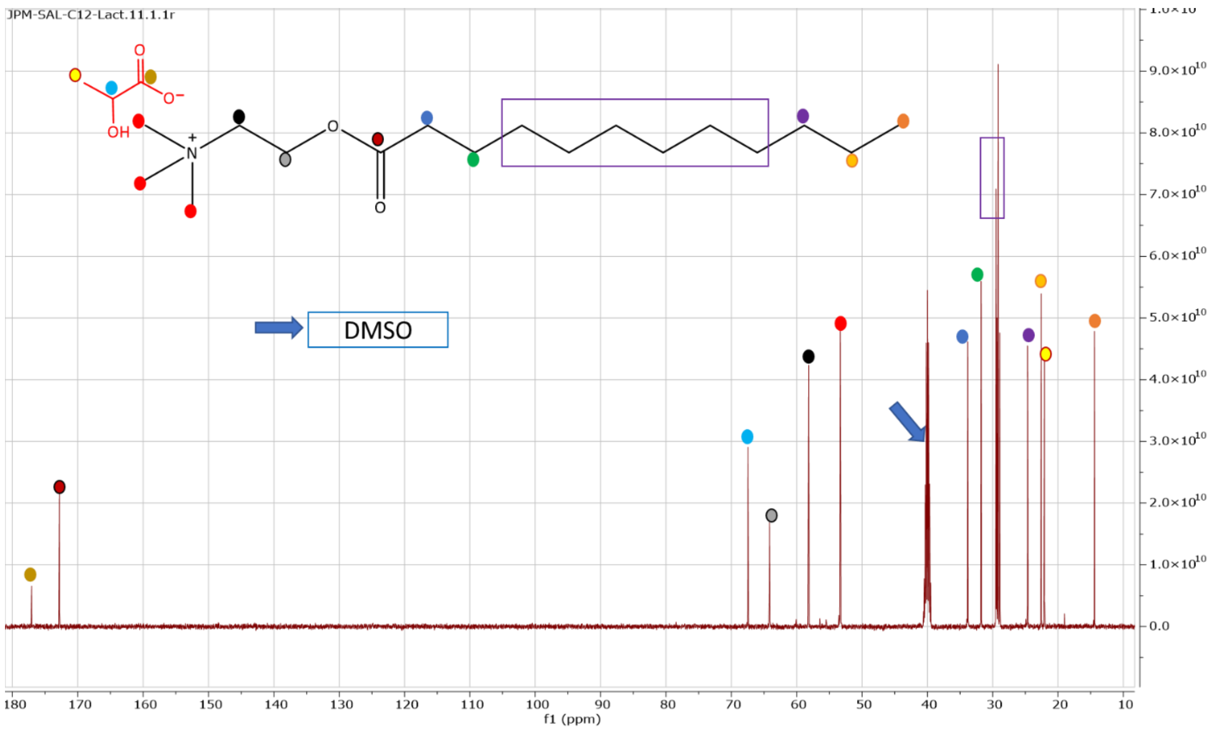

Figure S17: ^{13}C NMR spectrum of the IL Chol-C12-Lact

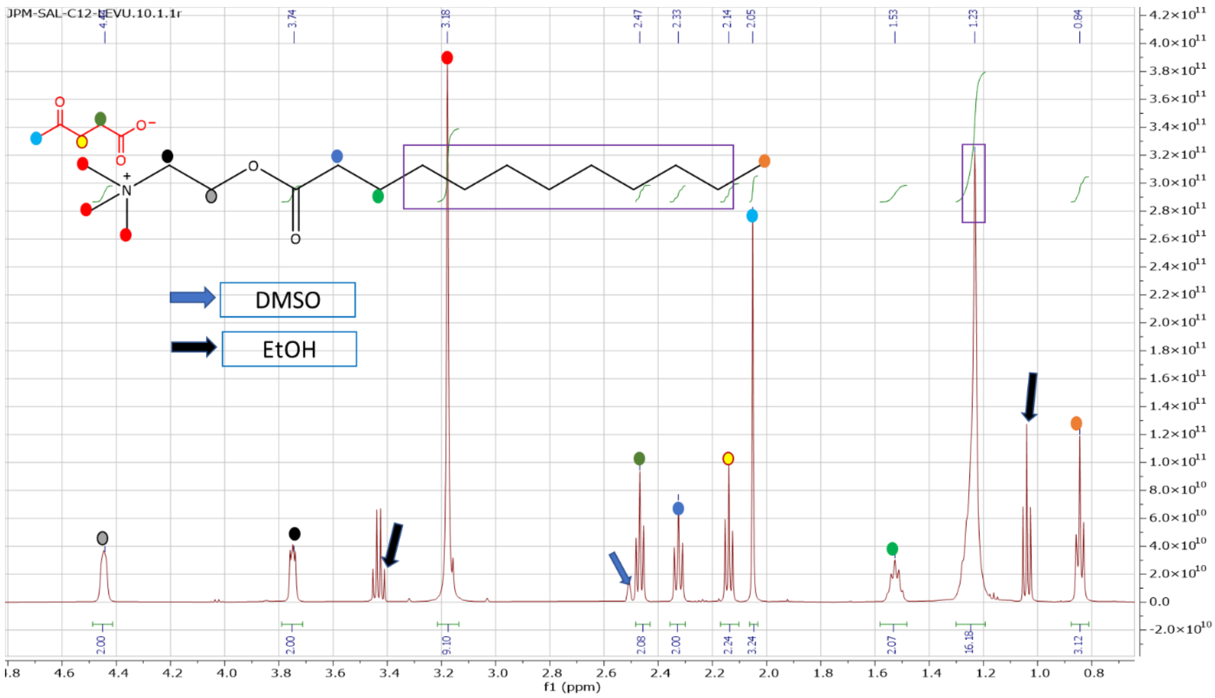

Figure S18: ^{1}H NMR spectrum of IL Chol-C12-Lev

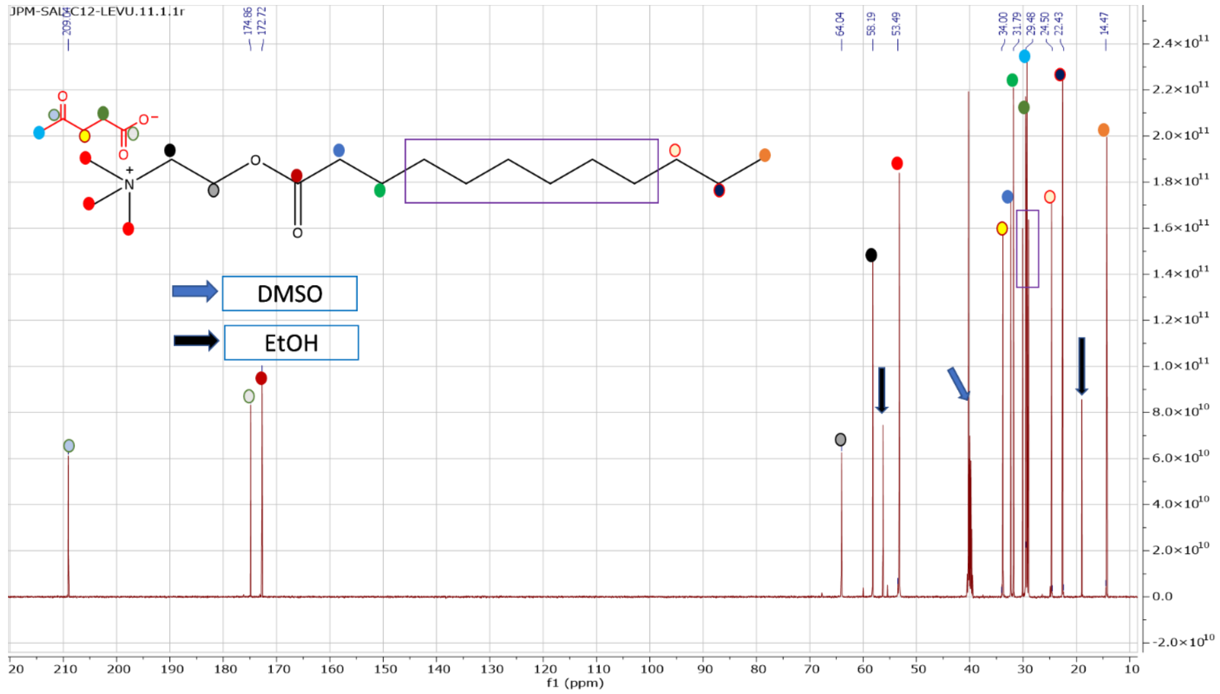

Figure S19: ^{13}C NMR spectrum of the IL Chol-C12-Lev

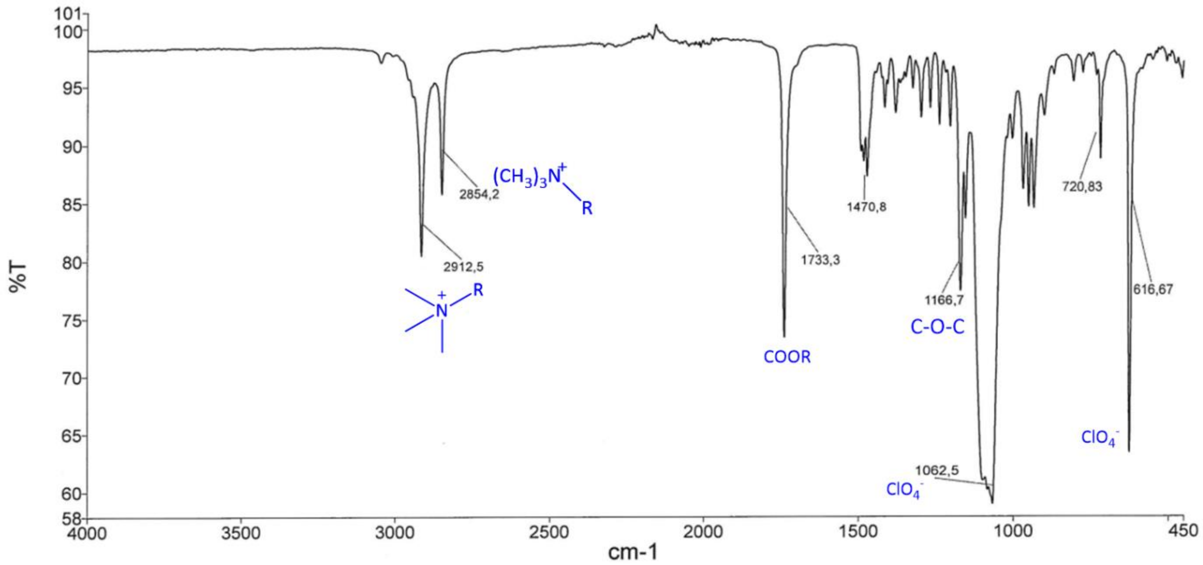

Figure S20: IR spectrum of the ester Chol-C12-ClO_4

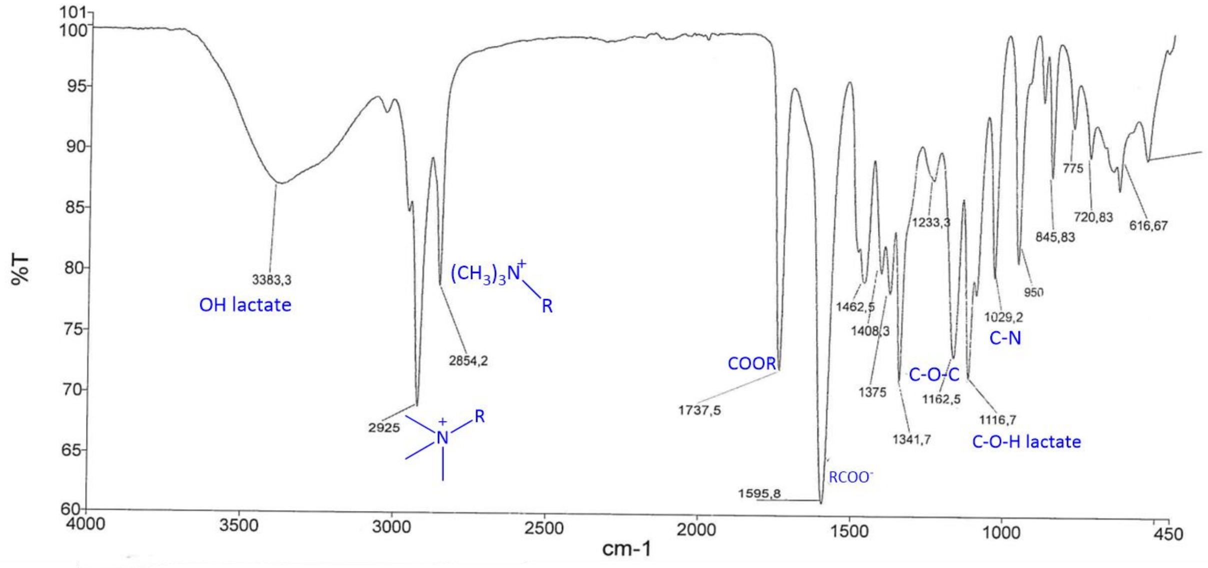

Figure S21: IR spectrum of the ILChol-C12-Lac

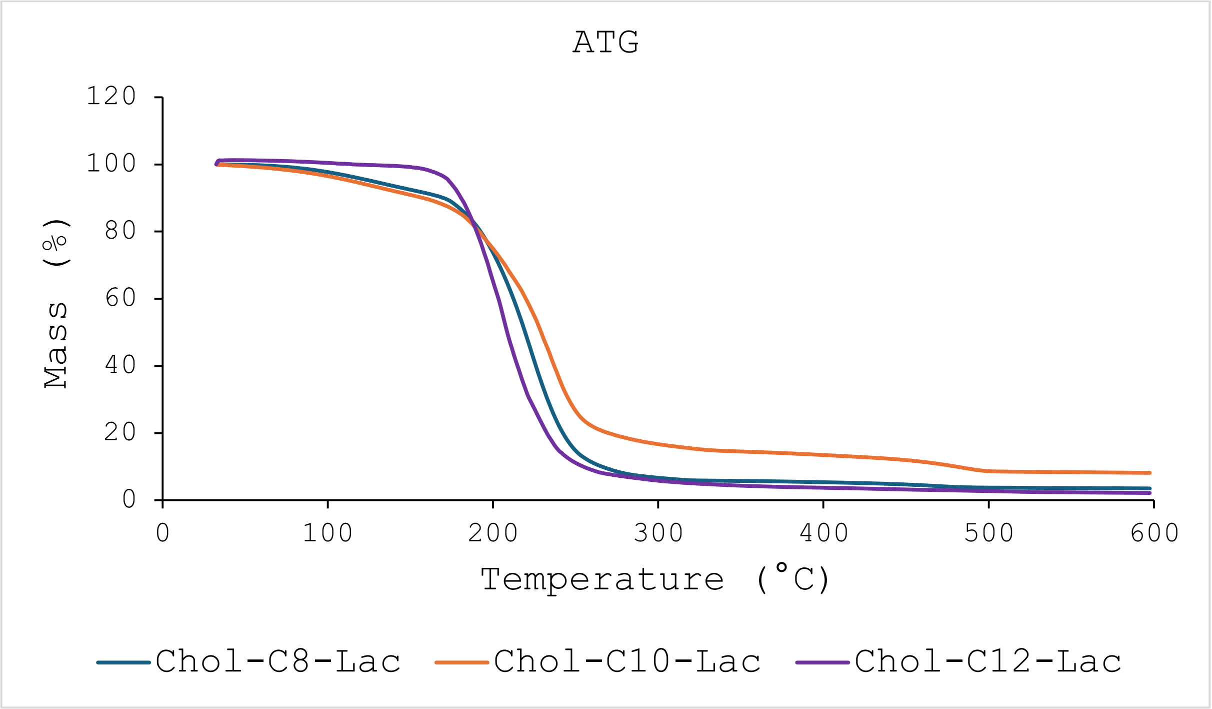

Figure S22: Thermo-gravimetric analysis (TGA) curves for ionic liquids

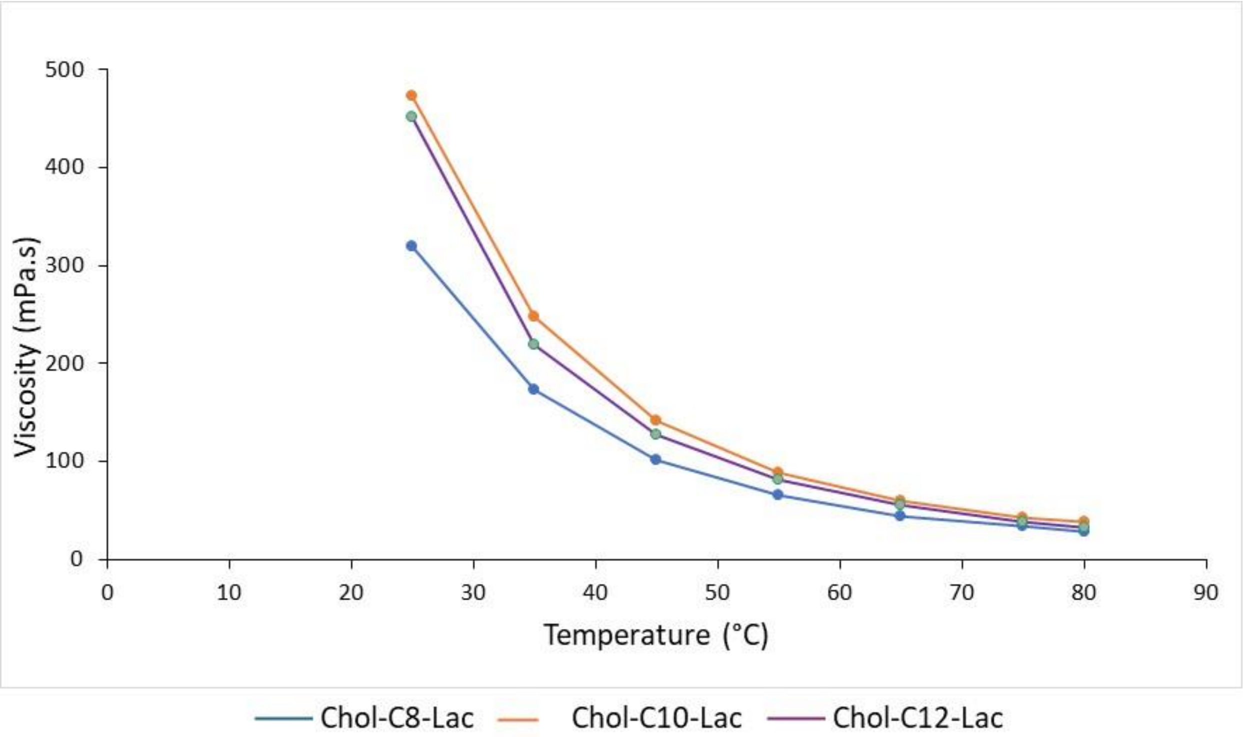

Figure S23: Viscosity of ionic liquids
